# Impact of delayed and prolonged fixation on the evaluation of immunohistochemical staining on lung carcinoma resection specimen

**DOI:** 10.1007/s00428-019-02595-9

**Published:** 2019-07-01

**Authors:** Maartje van Seijen, Luka Brcic, Atilio Navarro Gonzales, Irene Sansano, Matyas Bendek, Iva Brcic, Birgit Lissenberg-Witte, H. Ibrahim Korkmaz, Thomas Geiger, Rosita Kammler, Rolf Stahel, Erik Thunnissen, On behalf of ETOP

**Affiliations:** 1 Department of Pathology, Amsterdam University Medical Centers, VU University Medical Center, Amsterdam, The Netherlands; 2 Present Address: Department of Molecular Pathology, The Netherlands Cancer Institute, Amsterdam, The Netherlands; 3 Institute of Pathology, Medical University of Graz, Graz, Austria; 4 Valencia Hospital General Universitario, Valencia, Spain; 5 Vall d’Hebron University Hospital Barcelona, Spain, Barcelona, Spain; 6 National Institute of Pulmonary Medicine Budapest, Budapest, Hungary; 7 Present Address: Department of Pathology, Karolinska University Hospital, Stockholm, Sweden; 8 Department of Epidemiology and Biostatistics, Amsterdam UMC, VU University Medical Center, Amsterdam, The Netherlands; 9 ETOP Bern, Bern, Switzerland; 10 University Hospital of Zurich, Zurich, Switzerland

**Keywords:** Pre-analytical, Fixation, Immunohistochemistry, Lung adenocarcinoma

## Abstract

Pre-analytical factors, such as fixation time, influence morphology of diagnostic and predictive immunohistochemical staining, which are increasingly used in the evaluation of lung cancer. Our aim was to investigate if variations in fixation time influence the outcome of immunohistochemical staining in lung cancer. From lung resections, specimen with tumor size bigger than 4 cm, 10 samples were obtained: 2 were put through the standard fixation protocol, 5 through the delayed, and 3 through the prolonged fixation protocol. After paraffin embedding, tissue microarrays (TMAs) were made. They were stained with 20 antibodies and scored for quality and intensity of staining. Samples with delay in fixation showed loss of TMA cores on glass slides and deterioration of tissue quality leading to reduction in the expression of CK 7, Keratin MNF116, CAM 5.2, CK 5/6, TTF-1, C-MET, Napsin A, D2-40, and PD-L1. Prolonged fixation had no influence on the performance of immunohistochemical stains. Delay of fixation negatively affects the expression of different immunohistochemical markers, influencing diagnostic (cytokeratins) and predictive (PD-L1) testing. These results emphasize the need for adequate fixation of resection specimen.

**Electronic supplementary material:**

The online version of this article (10.1007/s00428-019-02595-9) contains supplementary material, which is available to authorized users.

## Introduction

Tissue samples are used for diagnostic and predictive analysis. Fixation process, preventing degradation, is essential in preserving the tissue. Commonly used fixative is neutral buffered formalin. However, there is a long list of variables in tissue handling, highly varying around the world [1], from the moment tissue is removed from a patient to formalin fixation and from formalin fixation to paraffin embedded (FFPE) tissue [2], influencing tissue quality. If the time between collection of tissue and actual formalin fixation is too long, tissue will degrade, morphology will deteriorate, and morphologic evaluation by pathologists will be hampered.

In the last 3–4 decades, immunohistochemistry (IHC) has become an essential procedure in pathology diagnostics [3–5]. It is important for tumor classification and characterization of underlying agents in many infectious diseases [6] and, in addition, is also increasingly used in predictive testing [4]. IHC involves the process of antibody binding to an epitope, i.e., part of a protein. For this binding proper, three-dimensional structure of the epitope is crucial. “Time before fixation,” as a very important pre-analytical factor, influences degradation of proteins and therefore may cause reduced binding of the primary antibody. It has already been shown in breast cancer that delay in fixation influences the outcome of immunohistochemical staining [7, 8].

For reproducible immunohistochemical staining evaluation in lung cancer, it is important to know if, and how, pre-analytical factors influence the expression of different diagnostic antibodies in a daily diagnostic setting. In biopsies, the samples are usually immediately placed in fixative, while for resection specimen, there is usually a delay.

The aim of this study was to investigate the influence of delayed and prolonged fixation on immunohistochemical evaluation in resection specimen of non-small cell lung carcinoma (NSCLC).

## Material and methods

### Tissue selection

In lung resection specimens with NSCLC bigger than > 4 cm, tumor tissue was collected at pathology departments in four institutes (Medical University of Graz, Austria; Vall d’ Hebron University Hospital Barcelona, Barcelona, Valencia Hospital General Universitario, Valencia, Spain; National Institute of Pulmonary Medicine Budapest, Hungary). Besides tissue collection for routine diagnostic procedures, ten additional tumor samples of approximately equal size (0.5 × 0.5 × 0.3 cm) were taken and put through different fixation protocols (as shown in Table 1), always on room temperature. Ten blocks per case and two cores per block were collected in total. Ischemia time, and the transport time of the unfixed samples to the pathology department, was recorded. Fixation was carried out in 10% neutral buffered formalin with PBS (approximately 4% formaldehyde *w*/*v*).

**Table 1:** Applied variations in time for delay in fixation and prolonged fixation, both at room temperature. Note that samples 1 and 2 received standard fixation

Sample no.	Delay in fixation	Fixation time
1, 2	0 h	24 h
3	0 h	2 days
4	0 h	4 days
5	0 h	7 days
6	1 h	24 h
7	6 h	18 h
8	24 h	24 h
9	48 h	24 h
10	96 h	24 h

### Tissue microarray

For the construction of tissue microarrays (TMAs), two 1-mm cores were taken from each of the collected samples and embedded in paraffin blocks. A separate TMA block was constructed for prolonged fixation and for delay in fixation, each included samples with standard fixation (sample 1 or 2). Histologic sections (3-μm thick) of the TMA blocks were cut for further IHC.

### Immunohistochemical markers

The following immunohistochemical markers were evaluated: cytokeratins (CK) AE1/AE3, CK 7 (Monosan and Dako), CAM 5.2, KER MNF-116, CK 5/6, p40, p80, p63, thyroid transcription factor (TTF)-1 (Ventana and Dako), BRAFV600E, C-MET, ROS1, ALK (D5F3), EGFR (Dako), Napsin A, D2-40, programmed cell death protein 1 (PD-L1) (22c3 and E1L3N (XP)), synaptophysin, chromogranin A, and CD 56. Details about the antibodies and procedure including retrieval methods for each are shown in supplementary Table 1.

Five pathologists scored independently the intensity of staining of both tumor and normal tissue (if present) in the laboratory where IHC was performed. Negative staining (< 1% of the cells stained) was scored as 1. Positive staining was graded by intensity of staining independent of the number of cells which were stained (except for PD–L1, see below) and scored as 2–4: 2—weak, defined as visible staining at microscope objective × 40 magnification; 4—strong, defined as visible staining at microscope objective × 2.5–4; and 3—moderate, for objectives within between magnification (Table 2). If individual cells were difficult to distinguish, or if the difference between normal cells and tumor cells was difficult to distinguish (often resulting in stromal staining and corresponding with non-interpretable slides), it was regarded as poor tissue quality and scored as 5. In the event that there was no core, no normal, or no tumor tissue present, scores of 0, 6, and 7 were assigned, respectively (Table 2). For PD-L1, the cell membrane staining was evaluated and expressed as a percentage of positive tumor cells in 5 categories (< 1%, 1–5, 5–10, 10–25, 25–50, and > 50%).

**Table 2:** Scoring system of immunohistochemistry on the TMAs

Scores	Explanation
0	= No core present
1	= Negative
2	= Weak positive (+)
3	= Moderate positive (++)
4	= Strong positive (+++)
5	= Poor quality of tissue (difficult to differentiate between tumor and normal tissue)
6	= No tumor present
7	= No normal tissue present

### Data analysis

IHC was scored in tumor and normal epithelium, if present. Separate comparisons were performed for standard fixation versus delayed/prolonged fixation distributed over normal and tumor tissue for each IHC marker with respect to (i) loss of TMA cores (dichotomized as score 0 vs score 1–5), (ii) within the available cores distinction of poor quality TMA cores versus cores with sufficient IHC quality for scoring intensity of staining (dichotomized as score 1–4 vs score 5), and (iii) within the cores with sufficient quality the intensity of IHC staining was scored. “Availability of a core” was defined as a core present on the glass slide. If a core was present in the paraffin block, but not in the corresponding histologic section, the core was not available (i.e., score 0). For (ii), also the score for all IHC combined was calculated. For the analysis per tissue block, the best score of the two duplicate cores was used.

McNemar and Wilcoxon signed rank test were used to compare the scores between the different samples of the diverse prolonged and delayed fixation times. Statistical analyses were performed with SPSS version 22 (IBM Corp., Armonk, NY, USA). Significance level was set at 0.05; *p* values < 0.1 were considered to indicate a statistical trend.

## Results

### Number of TMA cores

In total, 20 NSCLC tumor samples were used for this study. From the total number of 400 cores (10 blocks/case and 2 cores/block), 84% contained tumor tissue, and in 67% of the cores, also normal bronchial epithelial tissue was present.

From the expected 400 cores per IHC stain, 27% and 35% of the cores did not stick on the glass slide for prolonged and delayed fixation respectively. This resulted in 73% and 65% available cores to examine for IHC. The number of cores in prolonged/delayed fixation compared with the number in standard fixation is shown in Table 3. For example, CK 7 (Monosan) in delayed fixation showed loss of tissue cores (*p* < 0.01) compared to the standard fixation in normal tissue. Also, CK 7 (Dako), CAM 5.2, TTF-1 (2×), p40, p63, chromogranin A, CD 56, BRAFV600E, EGFR, ROS1, C-MET, p80, and PD-L1 were less cores present on the IHC slides than in the standard fixation group compared to 24 h (or more) delay in fixation (see Table 3, and for more detailed information, see supplementary Table 2). The prolonged fixation did not show loss of cores on slides in comparison with standard fixation.

**Table 3:** Data of standard fixation was compared to delayed and prolonged fixation for “Number of cores,” “quality of available cores,” and “IHC staining intensity in good quality cores” in the different stains. A downward arrow for number of cores denotes loss in the slide compared to the paraffin block

Antibody	Number of cores				Quality of available cores				IHC staining in sufficient quality cores			
Fixation	Delayed		Prolonged		Delayed		Prolonged		Delayed		Prolonged	
	N	T	N	T	N	T	N	T	N	T	N	T
CK 7 (Monosan)	↓**	=	↑*	=	=	=	=	=	=	↓	=	=
CK7 (Dako)	=	↓*	=	=	=	=	=	=	=	↓	=	↓
Ker MNF116	=	=	=	=	↓*	=	=	=	=	↓*	=	=
AE1/AE3	=	=	=	=	=	↓**	=	=	=	=	=	=
CAM 5.2	=	↓**	=	=	=	=	=	=	=	↓	↓	=
CK 5/6	=	=	=	=	=	=	=	=	↓	=	=	=
TTF-1	=	↓*	=	=	=	=	=	=	=	↓	=	=
TTF-1 (Ventana)	↓	↓*	=	=	=	=	=	=	=	↓*	=	=
TTF-1 (Dako)	=	=	=	=	=	=	=	=	=	=	=	=
p40	↓	↓**	=	=	=	=	=	=	=	=	=	=
p63	=	↓**	=	=	=	=	=	=	=	=	=	=
D2-40	=	=	=	=	=	=	=	=	↓*	=	=	=
Synaptophysin	=	=	=	=	↓**	↓**	=	=	=	=	=	=
Chromogranin A	↓*	↓*	=	=	=	↓	=	=	=	=	=	=
CD 56	↓	↓	=	=	↓*	↓*	=	=	=	=	=	=
Napsin A	=	=	=	=	=	=	=	=	↓*	↓*	=	↓
BRAFV600E	=	↓*	=	=	=	=	=	=	NoS	NoS	NoS	NoS
EGFR	=	↓	=	=	=	=	=	=	=	=	=	=
ROS1	=	↓*	=	=	=	=	=	=	NoS	NoS	NoS	NoS
C-MET	↓*	↓**	=	=	=	=	=	=	↓*	↓*	=	=
ALK D5F3	=	=	=	=	=	=	=	=	NoS	NoS	NoS	NoS
p80	=	↓	↑*	=	=	=	=	=	NoS	NoS	NoS	NoS
PD-L1 (E1L3N (XP))	=	↓*	=	=	=	=	=	=	↓**	↓	=	=
PD-L1 (22c3)	=	↓*	=	=	=	=	=	=	=	=	=	=

↓or↑—If any of the comparisons showed a downward or upward trend (*p* < 0.10), an arrow was assigned respectively

*N* normal respiratory epithelium, *T* tumor, *=* no effect, *NoS* no staining: tumor or normal respiratory epithelium is negative for this antibody in standard fixation

*p < 0.05; **p < 0.01

### Tissue morphological quality of TMA cores

**Fig. 1:**
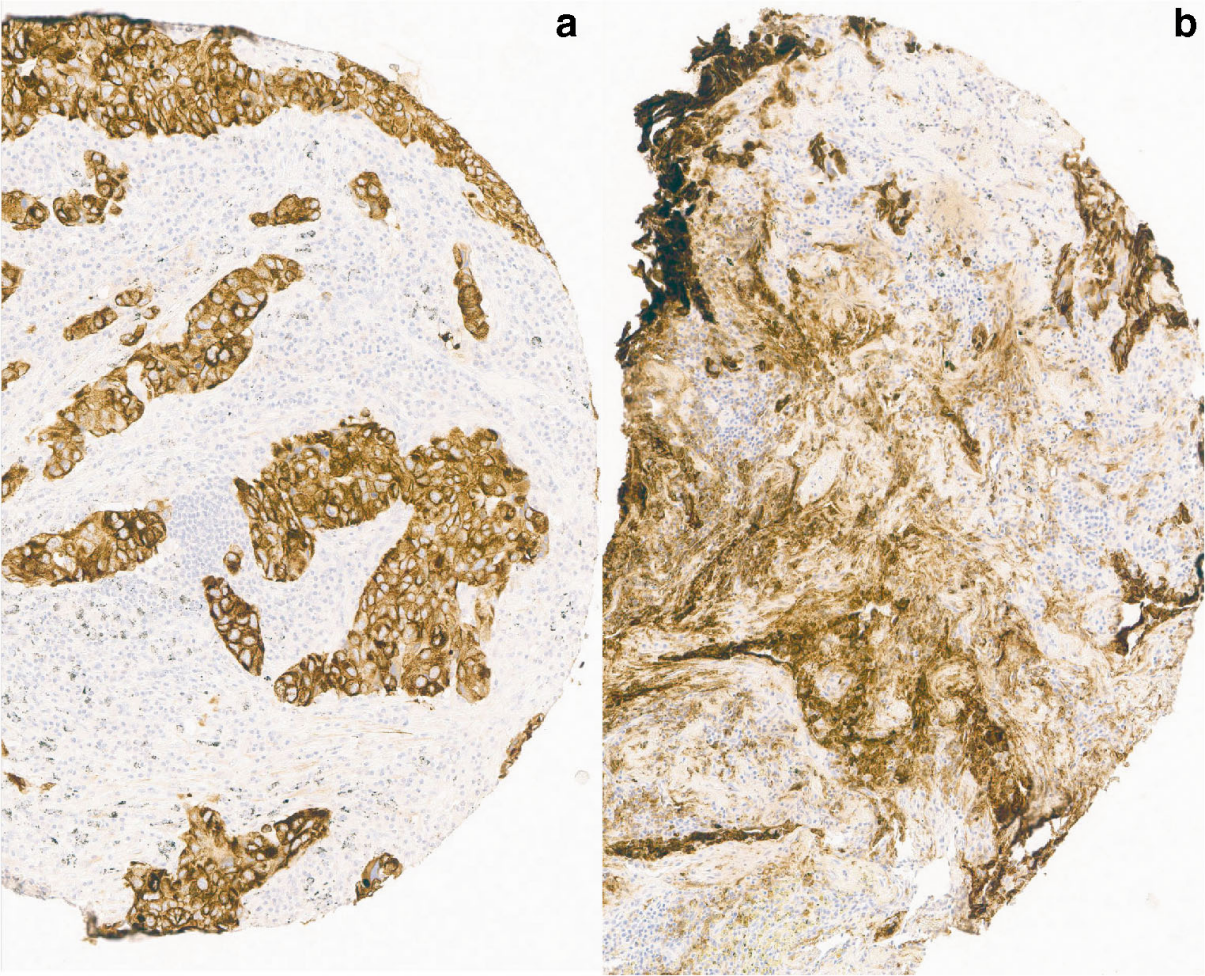
Example of a CK 7 (Monosan) staining after normal fixation (**a**) and after 96 h delay in fixation (**b**). A is scored as sufficient and B as insufficient quality, due to prominent non-specific (background) staining (for both objective × 15)

For the available cores on the slide, we estimated the quality of the cores for standard, delayed, and prolonged fixation for each antibody. The samples with an available IHC score for intensity (score 1–4) were compared to the alternative: poor quality (score 5) corresponding to non-interpretable staining. Figure 1 shows an example of a TMA scored as poor quality. As shown in Table 3, immunohistochemical stains keratin MNF116, AE1/AE3, synaptophysin, chromogranin A, and CD 56 demonstrated statistically significant reduction in quality in delayed fixation samples compared to standard fixation.

In standardly fixed samples, a score 5 (poor quality) was almost never assigned, in contrast to samples in delayed and prolonged fixation groups (for more in detailed information, see supplementary Table 3). For all antibodies combined, a significant decrease in quality is shown for 1, 24, 48, and 96 h of delay, compared to standard fixation (Table 4). The prolonged fixation group did not demonstrate major differences in tissue quality compared to the standard fixation group (supplementary Table 3).

**Table 4:** Presentation of the “quality of available cores” for all antibodies combined, in standard fixation compared to delayed fixation

Fixation delay (h)	Histology		Standard fixation		*p* value
			Evaluable	Poor quality	
1	Normal	Evaluable	184	7	*0.029*
		Poor quality	19	3	
	Tumor	Evaluable	302	9	*< 0.001*
		Poor quality	33	3	
6	Normal	Evaluable	186	7	0.63
		Poor quality	10	2	
	Tumor	Evaluable	354	10	0.68
		Poor quality	13	2	
24	Normal	Evaluable	157	2	*< 0.001*
		Poor quality	40	6	
	Tumor	Evaluable	285	2	*< 0.001*
		Poor quality	48	6	
48	Normal	Evaluable	173	7	*0.029*
		Poor quality	19	5	
	Tumor	Evaluable	301	8	*< 0.001*
		Poor quality	32	4	
96	Normal	Evaluable	116	1	*< 0.001*
		Poor quality	52	5	
	Tumor	Evaluable	233	1	*< 0.001*
		Poor quality	69	4	

### IHC staining intensity

For the cores with a staining intensity score (negative, positive +, ++, +++, scores 1–4 respectively), we compared the staining intensity (independent of the number of stained cells) of delayed and prolonged fixation to standard fixation. The following antibodies: CK 7 (Dako), CK 7 (Monosan), Ker MNF116, CAM 5.2, CK 5/6, TTF-1, C-MET, Napsin A, D2-40, TTF-1 (Ventana), and PD-L1 (22C3 and E1L3N (XP)) showed a significant decrease in intensity of staining (Table 3). Intensity of IHC staining decreases from 24 h of delay onwards (supplementary Table 4).

### PD-L1 staining

**Fig. 2:**
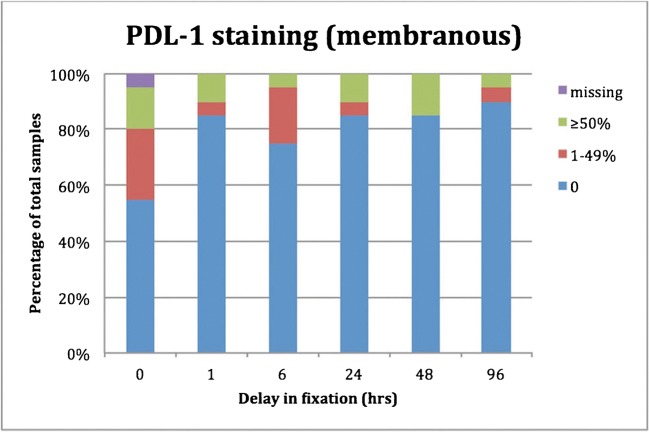
The distribution of PD-L1 (E1L3N (XP)) staining divided in 4 categories is shown for samples with delay in fixation. Of note, the number of cases with positive PD-L1 staining (1–49% and ≥ 50%) is lower after delay in fixation

**Fig. 3:**
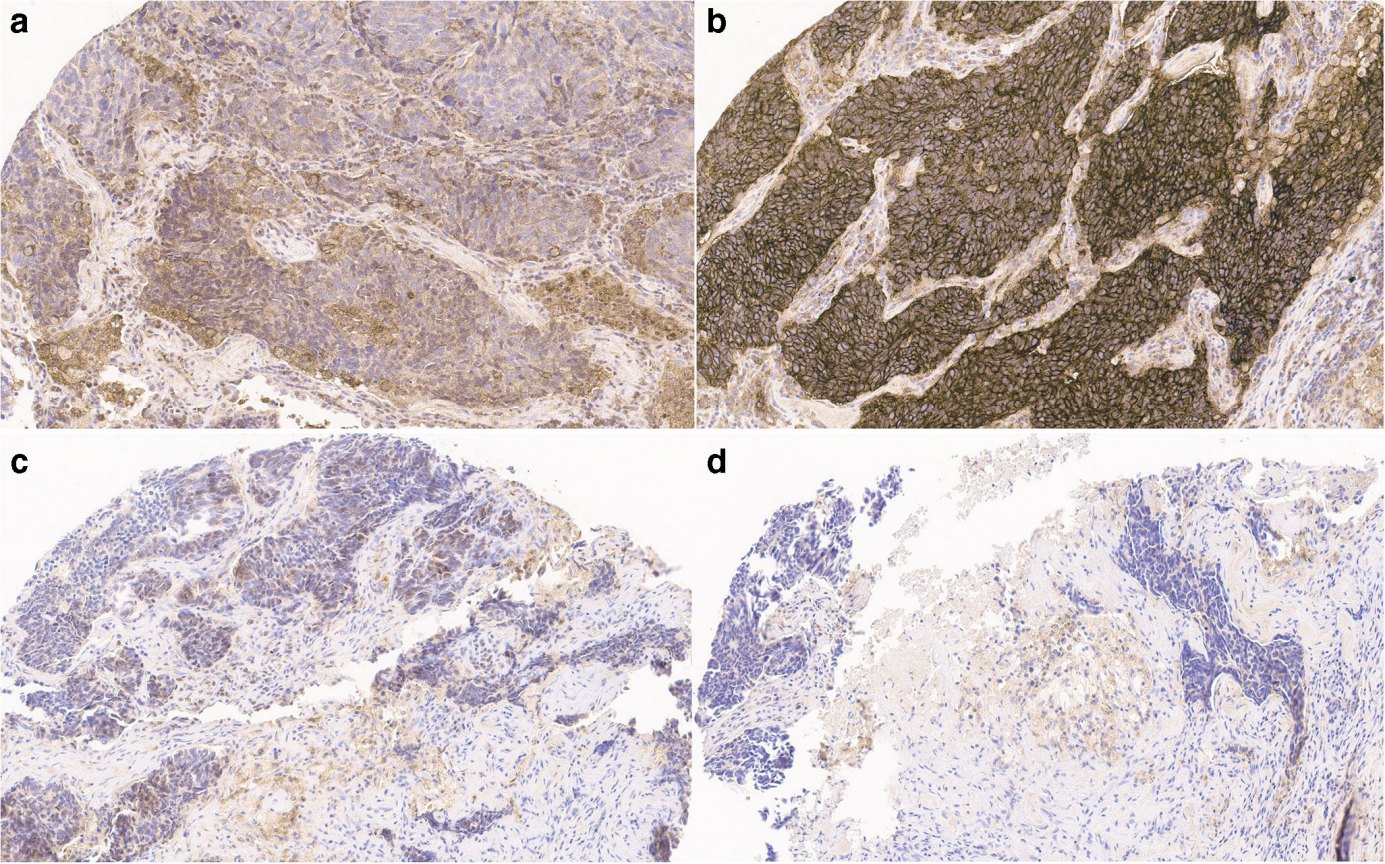
Presentation of PD-L1 staining in a tumor sample after normal fixation (**a**), after 6 h (**b**), 48 h (**c**), and 96 h (**d**) of delay in fixation (for all objective × 20). Note: deterioration of membrane staining in 48+ h delayed fixation and increase of non-specific staining

The cores of the delayed fixation group showed a reduction in percentage of tumor cells with positive membrane staining compared to standard fixation (Fig. 2). This effect is already shown after one hour in delay and is more pronounced with increasing time of delay in fixation (see Fig. 3 for an example). The samples with extended fixation do not show a change in PD-L1 staining (Table 3; supplementary Table 5).

## Discussion

From the pre-analytical variables studied on lung cancer tissue, delay in fixation has a detrimental effect on the number of available cores present on the slides, morphological quality, and IHC staining intensity. This effect was shown for both diagnostic (intensity of staining) and predictive (intensity and percentage of stained cells) markers. In contrast, prolonged fixation time has minor effect on the core number, tissue quality, and the evaluation of IHC staining.

The remarkable reduction of availability of expected cores on the slide denotes a relevant effect of tissue preservation during cutting of the paraffin section and sticking of the cores to the glass slide. This is in line with a recent study of Lundström and colleagues, who found on average a loss of 14% [9]. Furthermore, they observed that loss of cores was influenced by characteristics of tissue and pre-treatments [9].

Effect of pre-analytical variables on IHC has been described in the past decade^,^ [1, 10, 11]. The effect of delay in fixation is dependent on protein half-life. For example, phosphoproteins are quite labile and epitope degradation can happen within 30 min of cold ischemia time [12]. Although information on protein half-life is limited, indirect information is obtained in studies performed with immunohistochemical markers for breast cancer, i.e., estrogen receptor (ER), progesterone receptor (PR), and human epidermal growth factor 2 (HER2). Khoury et al. had not found significant differences in the extent or intensity of immunostaining with a fixation delay of less than 8 h [13]. We have observed deterioration in intensity of IHC staining from 24 h of delay (supplementary Table 5), except for PDL-1 marker, where we have already shown a deteriorating effect after one hour of delay in fixation.

Practically, as also shown by our results, fixation should be started as soon as possible after removing the tissue from a patient, because a delay affects the whole downstream process of tissue handling. Table 5 shows an overview of pre-analytical factors that could influence immunohistochemical staining, including some factors that could cause delay in fixation. Penetration rate, size of the specimen, and tissue-fixative ratio could cause incomplete fixation causing cross-linking only at the periphery of the tissue block. The center remains raw (or coagulatively fixed by alcohol during the dehydration step later in the tissue-handling process) and not properly fixed, resulting in more intense staining of the center or just opposite in the periphery, depending on the antibody that was used [6]. Generally assumed is a penetration rate of formalin of 1 mm/h [15]. However, this may be lower as shown in an example in supplementary Figure 1. Here, the time between registration and cutting after fixation was more than 40 h for fixation of 8 mm tissue, yielding a diffusion rate of about 0.2 mm/h. The relevance of a proper fixation procedure on Ki-67 was shown in breast cancer, where specimens that had been cut before fixation had a higher labeling than specimens that had not been cut [16].

**Table 5:** Pre-analytical variables from sample collection till start of fixation modified from Bass et al.^1^ and Engel et al.^9^)

Pre-analytical variables	(Biological) consequence	References
Warm ischemia time	As soon as the blood supply is cut off, hypoxia, ischemia, and metabolic stress may occur and lead to protein degradation.	Thompson et al. [14]
Cold ischemia time	Defined as time after removal from the body until fixation. Delay in fixation may induce various changes in protein levels.	Thompson et al. [14]
Temperature	Delay of fixation at room temperature has a more pronounced effect than storage at 4C.	Thompson et al. [14]
Specimen size and density	A larger specimen requires more time for proper fixation.	
Penetration rate	Generally assumed is a penetration rate of formalin of 1 mm/h. (Supplementary Figure 1 in this study 0.2 mm/h).	Howat et al. [12]
Fixative preparation	Formaldehyde is stable after 24 h. Polymerization slightly decreases the concentration during preparation.	
Tissue to fixative ratio	Tissue to fixative ratio could influence the time of fixation	
Decalcification	Variability in decalcification protocols and in preservation of antigen is reported.	Fitzgibbons et al. [8]
Protein, RNA, DNA half life	Phosphoproteins have short half-life. Most other unknown.	Vassilakopoulou et al. [10]

Prolonged fixation results in increasing cross-links [6]. However, it was shown that epitope retrieval methods could diminish or even abolish the cross-links and the negative effect on IHC [17]. The improved epitope retrieval methods likely explain why we did not show an effect of prolonged fixation on evaluation of IHC. This is in line with an animal model performed by Webster et al., in which they have shown no effect of prolonged fixation for example for chromogranin A and cytokeratin AE1/AE3. On the other hand, they have shown a decrease in immunoreactivity for CK 7 after 3 days of fixation, which is in contrast to our results [18]. In daily practice, more cross-links are generated during the weekends, when the laboratories are closed and tissue remains immersed in formalin fixative than during weekdays. According to our results for the tested antibodies, combined with the epitope retrieval methods, this prolonged fixation has no diagnostic implication.

Guidelines exist for analytic validation of immunohistochemical tests before their application to patient specimens [10]. Recently, information on clinical validation of PD-L1 was described [14]. However, these guidelines and cell block studies on PD-L1 [19, 20] do not contain specific information about pre-analytical factors, although these factors could have effects on the IHC test outcome. To our knowledge, this is the first study showing reduction of PD-L1 staining due to delay in fixation.

For breast cancer in the USA, the American Society of Clinical Oncologists (ASCO) and the College of American Pathologists (CAP) recommend limiting cold ischemic time to 60 min or less for ER testing [21]. Our results clearly demonstrate that it is important to standardize specimen handling and give recommendations to minimize the effect of pre-analytical variables.

Our study has several limitations: (i) the representation of the cores, where the aim was collecting tumor samples, and some of the cores instead contained post-obstruction pneumonia, mimicking tumor at gross examination; (ii) as we primarily aimed for tumor sampling, not all cores also contained normal respiratory epithelium and some heterogeneity could have played a role in the differences of staining; (iii) the prospectively selected cases were not suitable for all applied antibodies, for example, none of the tumors were ALK or BRAF V600E positive; (iv) although samples were stained in different laboratories and evaluated by corresponding pathologist, and similar antibodies revealed similar data, the effect of some interlaboratory and interobserver variability cannot be excluded. Color standardization may reduce variability [22]; (v) no minimum volume of formalin fixative was defined beforehand in the study protocol; (vi) as the antibody binding is epitope dependent, it is not excluded that diminishing of staining also holds for other epitopes of the same protein.

In conclusion, we showed that delay in fixation has influence on the amount of TMA cores on a glass slide and on the evaluation of immunohistochemical staining, reducing the staining intensity for some antibodies. The consequences of delay in fixation could diverge, leading from incorrect diagnosis to incorrect therapy indications. For that reason, resection specimens should be handled and fixed as soon as possible.

## Electronic supplementary material

ESM 1 (DOCX 27 kb)

ESM 2 (DOCX 110 kb)

ESM 3 (DOCX 112 kb)

ESM 4 (DOCX 64 kb)

ESM 5 (DOCX 19 kb)

ESM 6 (DOCX 1466 kb)

## References

[1] Bass BP, Engel KB, Greytak SR, Moore HM (2014). A review of preanalytical factors affecting molecular, protein, and morphological analysis of formalin-fixed, paraffin-embedded (FFPE) tissue: how well do you know your FFPE specimen?. Arch Pathol Lab Med.

[2] Neumeister VM (2014). Tools to assess tissue quality. Clin Biochem.

[3] Prinsen CFM, Klaassen CHW, Thunnissen FBJM (2003). Microarray as a model for quantitative visualization chemistry. Appl Immunohistochem Mol Morphol.

[4] Thunnissen E, Allen TC, Adam J, Aisner DL, Beasley MB, Borczuk AC, Cagle PT, Capelozzi VL, Cooper W, Hariri LP, Kern I, Lantuejoul S, Miller R, Mino-Kenudson M, Radonic T, Raparia K, Rekhtman N, Roy-Chowdhuri S, Russell P, Schneider F, Sholl LM, Tsao MS, Vivero M, Yatabe Y (2017) Immunohistochemistry of pulmonary biomarkers: a perspective from members of the pulmonary pathology society. Arch Pathol Lab Med arpa.2017-0138-SA. 10.5858/arpa.2017-0106-SA 10.5858/arpa.2017-0106-SA 28686497

[5] Torlakovic EE, Cheung CC, D’Arrigo C, Dietel M, Francis GD, Gilks CB, Hall JA, Hornick JL, Ibrahim M, Marchetti A, Miller K, van Krieken JH, Nielsen S, Swanson PE, Vyberg M, Zhou X, Taylor CR, From the International Society for Immunohistochemistry and Molecular Morphology (ISIMM) and International Quality Network for Pathology (IQN Path) (2017). Evolution of quality assurance for clinical immunohistochemistry in the era of precision medicine. Part 3: technical validation of immunohistochemistry (IHC) assays in clinical IHC laboratories. Appl Immunohistochem Mol Morphol AIMM.

[6] Werner M, Chott A, Fabiano A, Battifora H (2000). Effect of formalin tissue fixation and processing on immunohistochemistry. Am J Surg Pathol.

[7] Apple S, Pucci R, Lowe AC, Shintaku I, Shapourifar-Tehrani S, Moatamed N (2011). The effect of delay in fixation, different fixatives, and duration of fixation in estrogen and progesterone receptor results in breast carcinoma. Am J Clin Pathol.

[8] Khoury T (2012). Delay to formalin fixation alters morphology and immunohistochemistry for breast carcinoma. Appl Immunohistochem Mol Morphol.

[9] Lundström Y, Stud M, Lundström P, Stud M, Popova SN, Lindblom RPF, Alafuzoff I, Sci M (2018) Detection of changes in Immunohistochemical stains caused by postmortem delay and fixation time. Appl Immunohistochem Mol Morphol AIMM 27:238-245. 10.1097/PAI.0000000000000658 10.1097/PAI.0000000000000658 29912765

[10] Patrick LF, Linda AB, Lisa AF, Alsabeh R, Regan SF, Jeffrey DG, Thomas SH, Karabakhtsian RG, Patti AL, Marolt MJ, Steven SS, Anthony TS, Swanson PE (2014). Principles of analytic validation of immunohistochemical assays: guideline from the College of American Pathologists Pathology and Laboratory Quality Center. Arch Pathol Lab Med.

[11] Engel KB, Moore HM (2011) Effects of preanalytical variables on the detection of proteins by immunohistochemistry in formalin-fixed, paraffin-embedded tissue. Arch Pathol Lab Med. 10.1043/2010-0702-RAIR.1 10.5858/2010-0702-RAIR.1 21526952

[12] Vassilakopoulou M, Parisi F, Siddiqui S, England AM, Zarella ER, Anagnostou V, Kluger Y, Hicks DG, Rimm DL, Neumeister VM (2014). Preanalytical variables and phosphoepitope expression in FFPE tissue: quantitative epitope assessment after variable cold ischemic time. Lab Investig.

[13] Khoury T, Sait S, Hwang H, Chandrasekhar R, Wilding G, Tan D, Kulkarni S (2009). Delay to formalin fixation effect on breast biomarkers. Mod Pathol.

[14] Thunnissen E, de Langen AJ, Smit EF (2017). PD-L1 IHC in NSCLC with a global and methodological perspective. Lung Cancer.

[15] Howat WJ, Wilson BA (2014). Tissue fixation and the effect of molecular fixatives on downstream staining procedures. Methods.

[16] Arima N, Nishimura R, Osako T, Nishiyama Y, Fujisue M, Okumura Y, Nakano M, Tashima R, Toyozumi Y (2016). The importance of tissue handling of surgically removed breast cancer for an accurate assessment of the Ki-67 index. J Clin Pathol.

[17] von Wasielewski R, Werner M, Nolte M, Wilkens L, Georgii A (1994). Effects of antigen retrieval by microwave heating in formalin-fixed tissue sections on a broad panel of antibodies. Histochemistry.

[18] Webster JD, Miller MA, Dusold D, Ramos-Vara J (2009). Effects of prolonged formalin fixation on diagnostic immunohistochemistry in domestic animals. J Histochem Cytochem.

[19] Cree IA, Booton R, Cane P, Gosney J, Ibrahim M, Kerr K, Lal R, Lewanski C, Navani N, Nicholson AG, Nicolson M, Summers Y (2016). PD-L1 testing for lung cancer in the UK: recognizing the challenges for implementation. Histopathology.

[20] Lloyd IE, Zhou W, Witt BL, Chadwick BE (2017). Characterization of PD-L1 immunohistochemical expression in cell blocks with different specimen fixation and processing methods. Appl Immunohistochem Mol Morphol AIMM.

[21] Hammond MEH, Hayes DF, Dowsett M, Allred DC, Hagerty KL, Badve S, Fitzgibbons PL, Francis G, Goldstein NS, Hayes M, Hicks DG, Lester S, Love R, Mangu PB, McShane L, Miller K, Osborne CK, Paik S, Perlmutter J, Rhodes A, Sasano H, Schwartz JN, Sweep FCG, Taube S, Torlakovic EE, Valenstein P, Viale G, Visscher D, Wheeler T, Williams RB, Wittliff JL, Wolff AC (2010). American Society of Clinical Oncology/College of American Pathologists guideline recommendations for immunohistochemical testing of estrogen and progesterone receptors in breast cancer. J Clin Oncol.

[22] Fernandez-Carrobles MM, Bueno G (2016) Analysis of color standardization methods for the automatic quantification of IHC stain in breast TMA. Diagn Pathol:25–26. 10.17629/www.diagnosticpathology.eu-2016-8:220

