# Supplementary material for: Impact of delayed and prolonged fixation on the evaluation of immunohistochemical staining on lung carcinoma resection specimen
Source: Virchows Arch. 2019 Jul 1;475(2):191–9. doi: 10.1007/s00428-019-02595-9 (PMC6647403; doi:10.1007/s00428-019-02595-9)
Supplement: Supplementary file 1 — (DOCX 27 kb) [file 428_2019_2595_MOESM1_ESM.docx]

Supplementary table 1. Antibodies specifications and details of IHC procedure

| **Antibody** | **Company** | **Clone** | **Dilution** | **Incubation**  **time** | **Antigen retrieval method** | **Antigen retrieval incubation time** | **Detection method** | **Catalog number** |
| --- | --- | --- | --- | --- | --- | --- | --- | --- |
| *VUmc* |  |  |  |  |  |  |  |  |
| AE1/ AE3 | Dako | AE1/AE3 | 1/100 | 16 min | CC1 | 24 min | Optiview | M3515 |
| CK 7 | Monosan via Sanbio | OVTL12/30 | 1/100 | 32 min | CC1 | 24 min | Optiview | MON3014 |
| CAM 5.2 | Becton and Dickinson biosciences | Cam5.2 | 1/10 | 48 min | CC1 | 24 min | Optiview | 345779 |
| KER-MNF116 | Dako | MNF116 | 1/50 | 32 min | Protease 1 | 4 min | Optiview | M0821 |
| p40 | Biocare Medical | BC28 | 1/100 | 32 min | CC1 | 24 min | Optiview | ACI3066C |
| p63 | Immunologics (klinipath) | 4A4 | 1/600 in Dako reduc ab dil | 32 min | CC1 | 24 min | Optiview | ILM027611-C1 |
| TTF-1 | Dako | 8G7G3-1 | 1/800 in Dako reduc ab dil | 16 min | CC1 | 32 min | Optiview | M3575 |
| BRAFV600E | Springbiosciences | VE1 | 1/50 | 32 min | CC1 | 64 min | Optiview 2x12' | E19290 |
| C-MET | Ventana | SP44 | RTU | 16 min | CC1 | 64 min | UltraView DAB | 05571219001 |
| ROS1 | Cell Signaling Technology Inc | D4D6 | 1/50 | 48 min | CC1 | 32 min | OV+OV Ampl 4'x4' | 3287 |
| p80 | Monosan via Sanbio | 5A4 | 1/10 in Dako reduc ab dil | 32 min | CC1 | 48 min | OV+OV Ampl 4'x4' | MONX10639 |
| PD-L1 | Cell Signaling Technology Inc | E1L3N (XP) | 1/200 | 48 min | CC1 | 64 min | Optiview | 13684S |
| *Valencia Hospital General Universitario* |  |  |  |  |  |  |  |  |
| Napsin A | Master diagnóstica | BS10 | RTU | 15 min | CC1* | 20 min | MASTER POLYMER PLUS DETECTION SYSTEM (peroxidase) | MAD-000752-QD-10/N |
| D2-40 | Master diagnóstica | D2-40 | RTU | 10 min | CC1* | 20 min | MASTER POLYMER PLUS DETECTION SYSTEM (peroxidase) | MAD-000402-QD-10/N |
| CK 5/6 | Master diagnóstica | EP24/EP67 | RTU | 10 min | CC1* | 20 min | MASTER POLYMER PLUS DETECTION SYSTEM (peroxidase) | MAD-000651-QD-10/N |
| *Vall d'Hebron University Hospital Barcelona* |  |  |  |  |  |  |  |  |
| CD 56 | Ventana | MRQ-42 | RTU | 32 min | CC1 | 20 min | Ultraview DAB  + Ampification | 760-4596 |
| Chromogranin | Ventana | LK2H10 | RTU | 16 min | CC1 | 20 min | Ultraview DAB | 760-2519 |
| Synaptophysin | Ventana | MRQ-40 | RTU | 32 min | CC1 | 36 min | Ultraview DAB | 760-4595 |
| *Medical University of Graz* |  |  |  |  |  |  |  |  |
| TTF-1 | Ventana | 8G7G3-1 | RTU | 32 min |  |  | Ultraview DAB | 5269806001 |
| TTF-1 | Dako | 8G7G3-1 | 1:100 | 60 min | MW 9,0 | 40min (150Watt) | Envision DAB | K5007 |
| ALK | Ventana | D5F3 | RTU | 16 min | CC1 92min |  | Optiview DAB+Amplification | 760-700 760/099 |
| CK 7 | Dako | OV-TL12/0 | 1:100 | 20 min |  |  | +Protease Chemate AEC | K5001 K3464 |
| EGFR | Dako |  | RTU | 30 min |  |  | EGFR Kit DAB | K1492 |
| PD-L1 | Dako | 22C3 | 1:30 | 30 min | Omnis low pH | 50 min | Flex DAB | GV800 |
