# Supplementary material for: Impact of delayed and prolonged fixation on the evaluation of immunohistochemical staining on lung carcinoma resection specimen
Source: Virchows Arch. 2019 Jul 1;475(2):191–9. doi: 10.1007/s00428-019-02595-9 (PMC6647403; doi:10.1007/s00428-019-02595-9)
Supplement: Supplementary file 2 — (DOCX 110 kb) [file 428_2019_2595_MOESM2_ESM.docx]

Supplementary table 2 *Availability of cores in prolonged/delayed fixation compared with standard fixation*

| **Delay (hrs)** | **Tissue** | **Available** | **p80** | | | **CK 7 (Monosan)** | | | **Ker MNF 116** | | | **AE13** | | | **CAM 5.2** | | | **TTF-1 (Dako)** | | | **BRAFV600E** | | | **p40** | | |
| --- | --- | --- | --- | --- | --- | --- | --- | --- | --- | --- | --- | --- | --- | --- | --- | --- | --- | --- | --- | --- | --- | --- | --- | --- | --- | --- |
|  |  |  | **No** | **Yes** | **p** | **No** | **Yes** | **p** | **No** | **Yes** | **p** | **No** | **Yes** | **p** | **No** | **Yes** | **p** | **No** | **Yes** | **p** | **No** | **Yes** | **p** | **No** | **Yes** | **p** |
| 1 | normal | No | 8 | 6 | 0.75 | 1 | 4 | 0.38 | 4 | 5 | 1.00 | 3 | 4 | 0.39 | 7 | 2 | 0.45 | 8 | 3 | 1.00 | 11 | 4 | 1.00 | 10 | 4 | 1.00 |
|  |  | Yes | 4 | 2 |  | 1 | 14 |  | 5 | 6 |  | 8 | 5 |  | 5 | 6 |  | 3 | 6 |  | 3 | 2 |  | 3 | 3 |  |
|  | tumor | No | 0 | 7 | 0.070 | 0 | 6 | - | 0 | 2 | - | 0 | 2 | - | 0 | 4 | 0.38 | 0 | 5 | 0.45 | 0 | 6 | 0.13 | 0 | 6 | 0.13 |
|  |  | Yes | 1 | 12 |  | 0 | 14 |  | 0 | 18 |  | 0 | 18 |  | 1 | 15 |  | 2 | 13 |  | 1 | 13 |  | 1 | 13 |  |
| 6 | normal | No | 5 | 5 | 0.77 | 2 | 4 | 0.13 | 5 | 7 | 0.55 | 5 | 5 | 1.00 | 8 | 5 | 1.00 | 7 | 6 | 0.75 | 12 | 5 | 0.45 | 9 | 4 | 1.00 |
|  |  | Yes | 7 | 3 |  | 0 | 14 |  | 4 | 4 |  | 6 | 4 |  | 4 | 3 |  | 4 | 3 |  | 2 | 1 |  | 4 | 3 |  |
|  | tumor | No | 0 | 3 | 0.63 | 0 | 3 | - | 0 | 3 | - | 0 | 3 | - | 0 | 4 | 0.38 | 0 | 3 | 1.00 | 0 | 3 | 0.63 | 0 | 4 | 0.38 |
|  |  | Yes | 1 | 16 |  | 0 | 17 |  | 0 | 17 |  | 0 | 17 |  | 1 | 15 |  | 2 | 15 |  | 1 | 16 |  | 1 | 15 |  |
| 24 | normal | No | 7 | 3 | 0.73 | 2 | 12 | **<0.001** | 8 | 4 | 0.38 | 7 | 4 | 1.00 | 11 | 2 | 1.00 | 10 | 4 | 0.38 | 12 | 4 | 0.69 | 12 | 4 | 0.38 |
|  |  | Yes | 5 | 5 |  | 0 | 6 |  | 1 | 7 |  | 4 | 5 |  | 1 | 6 |  | 1 | 5 |  | 2 | 2 |  | 1 | 3 |  |
|  | tumor | No | 1 | 3 | 0.25 | 0 | 10 | - | 0 | 6 | - | 0 | 4 | - | 1 | 6 | **0.031** | 2 | 4 | 0.13 | 1 | 6 | **0.031** | 1 | 8 | **0.008** |
|  |  | Yes | 0 | 16 |  | 0 | 10 |  | 0 | 14 |  | 0 | 16 |  | 0 | 13 |  | 0 | 14 |  | 0 | 13 |  | 0 | 11 |  |
| 48 | normal | No | 6 | 3 | 0.51 | 1 | 9 | **0.021** | 7 | 6 | 0.29 | 2 | 6 | 0.61 | 9 | 4 | 1.00 | 8 | 6 | 0.51 | 11 | 4 | 1.00 | 10 | 4 | 1.00 |
|  |  | Yes | 6 | 5 |  | 1 | 9 |  | 2 | 5 |  | 9 | 3 |  | 3 | 4 |  | 3 | 3 |  | 3 | 2 |  | 3 | 3 |  |
|  | tumor | No | 0 | 4 | 0.38 | 0 | 10 | - | 0 | 4 | - | 0 | 2 | - | 0 | 10 | **0.012** | 1 | 4 | 0.38 | 0 | 4 | 0.38 | 0 | 9 | **0.021** |
|  |  | Yes | 1 | 15 |  | 0 | 10 |  | 0 | 16 |  | 0 | 18 |  | 1 | 9 |  | 1 | 14 |  | 1 | 15 |  | 1 | 10 |  |
| 96 | normal | No | 4 | 3 | 0.23 | 2 | 10 | **0.002** | 7 | 3 | 1.00 | 2 | 6 | 0.61 | 11 | 5 | 0.22 | 8 | 9 | 0.15 | 12 | 3 | 1.00 | 12 | 7 | 0.07 |
|  |  | Yes | 8 | 5 |  | 0 | 8 |  | 2 | 7 |  | 9 | 3 |  | 1 | 3 |  | 3 | 0 |  | 2 | 3 |  | 1 | 0 |  |
|  | tumor | No | 0 | 5 | 0.22 | 0 | 10 | - | 0 | 3 | - | 0 | 4 | - | 1 | 10 | **0.002** | 1 | 8 | **0.039** | 1 | 4 | 0.13 | 1 | 14 | **<0.001** |
|  |  | Yes | 1 | 14 |  | 0 | 10 |  | 0 | 16 |  | 0 | 16 |  | 0 | 9 |  | 1 | 10 |  | 0 | 15 |  | 0 | 5 |  |

| **Delay (hrs)** | **Tissue** | **Available** | **PD-L1** | | | **ROS1** | | | **C-MET** | | | **p63** | | | **CK 5/6** | | | **Napsin A** | | | **D2-40** | | | **TTF-1 (Ventana)** | | |
| --- | --- | --- | --- | --- | --- | --- | --- | --- | --- | --- | --- | --- | --- | --- | --- | --- | --- | --- | --- | --- | --- | --- | --- | --- | --- | --- |
|  |  |  | **No** | **Yes** | **p** | **No** | **Yes** | **p** | **No** | **Yes** | **p** | **No** | **Yes** | **p** | **No** | **Yes** | **p** | **No** | **Yes** | **p** | **No** | **Yes** | **p** | **No** | **Yes** | **p** |
| 1 | normal | No | 0 | 3 | 0.63 | 7 | 5 | 1.00 | 8 | 4 | 1.00 | 15 | 1 | 0.38 | 0 | 0 | - | 0 | 0 | - | 0 | 0 | - | 5 | 4 | 1.00 |
|  |  | Yes | 1 | 16 |  | 5 | 3 |  | 3 | 5 |  | 4 | 0 |  | 0 | 20 |  | 0 | 20 |  | 0 | 20 |  | 5 | 6 |  |
|  | tumor | No | 0 | 6 | 0.29 | 0 | 7 | 0.07 | 0 | 6 | 0.13 | 0 | 6 | 0.13 | 0 | 0 | - | 0 | 1 | 1.00 | 0 | 1 | 1.00 | 0 | 5 | 0.22 |
|  |  | Yes | 2 | 12 |  | 1 | 12 |  | 1 | 13 |  | 1 | 13 |  | 0 | 20 |  | 1 | 18 |  | 1 | 18 |  | 1 | 14 |  |
| 6 | normal | No | 0 | 2 | 1.00 | 5 | 5 | 0.77 | 7 | 3 | 1.00 | 16 | 1 | 0.63 | 0 | 1 | - | 0 | 1 | - | 0 | 1 | - | 6 | 4 | 1.00 |
|  |  | Yes | 1 | 17 |  | 7 | 3 |  | 4 | 6 |  | 3 | 0 |  | 0 | 19 |  | 0 | 19 |  | 0 | 19 |  | 4 | 6 |  |
|  | tumor | No | 0 | 3 | 1.00 | 0 | 3 | 0.63 | 0 | 3 | 0.63 | 0 | 3 | 0.63 | 0 | 1 | - | 0 | 2 | 1.00 | 0 | 1 | 1.00 | 0 | 3 | 0.63 |
|  |  | Yes | 2 | 15 |  | 1 | 16 |  | 1 | 16 |  | 1 | 16 |  | 0 | 19 |  | 1 | 17 |  | 1 | 18 |  | 1 | 16 |  |
| 24 | normal | No | 1 | 4 | 0.13 | 6 | 4 | 0.75 | 11 | 6 | **0.031** | 16 | 1 | 0.63 | 0 | 1 | - | 0 | 0 | - | 0 | 0 | - | 10 | 5 | 0.06 |
|  |  | Yes | 0 | 15 |  | 6 | 4 |  | 0 | 3 |  | 3 | 0 |  | 0 | 19 |  | 0 | 20 |  | 0 | 20 |  | 0 | 5 |  |
|  | tumor | No | 2 | 7 | **0.016** | 0 | 3 | 0.63 | 1 | 7 | **0.016** | 1 | 3 | 0.25 | 0 | 3 | - | 1 | 1 | 1.00 | 1 | 1 | 1.00 | 1 | 6 | **0.031** |
|  |  | Yes | 0 | 11 |  | 1 | 16 |  | 0 | 12 |  | 0 | 16 |  | 0 | 17 |  | 0 | 18 |  | 0 | 18 |  | 0 | 13 |  |
| 48 | normal | No | 0 | 4 | 0.38 | 7 | 3 | 0.73 | 8 | 6 | 0.51 | 15 | 1 | 0.38 | 0 | 2 | - | 0 | 0 | - | 0 | 1 | - | 4 | 6 | 1.00 |
|  |  | Yes | 1 | 15 |  | 5 | 5 |  | 3 | 3 |  | 4 | 0 |  | 0 | 18 |  | 0 | 20 |  | 0 | 19 |  | 6 | 4 |  |
|  | tumor | No | 1 | 5 | 0.22 | 0 | 6 | 0.13 | 0 | 9 | **0.021** | 0 | 5 | 0.22 | 0 | 2 | - | 0 | 0 | - | 0 | 2 | 1.00 | 0 | 4 | 0.38 |
|  |  | Yes | 1 | 13 |  | 1 | 13 |  | 1 | 10 |  | 1 | 14 |  | 0 | 18 |  | 1 | 19 |  | 1 | 17 |  | 1 | 15 |  |
| 96 | normal | No | 1 | 2 | 0.50 | 8 | 3 | 1.00 | 9 | 8 | 0.11 | 15 | 1 | 0.38 | 0 | 4 | - | 0 | 0 | - | 0 | 5 | - | 7 | 5 | 0.73 |
|  |  | Yes | 0 | 17 |  | 4 | 4 |  | 2 | 1 |  | 4 | 0 |  | 0 | 16 |  | 0 | 20 |  | 0 | 15 |  | 3 | 5 |  |
|  | tumor | No | 1 | 3 | 0.63 | 1 | 6 | **0.031** | 1 | 8 | **0.008** | 1 | 10 | **0.002** | 0 | 4 | - | 0 | 1 | 1.00 | 1 | 3 | 0.25 | 0 | 2 | 1.00 |
|  |  | Yes | 1 | 15 |  | 0 | 13 |  | 0 | 11 |  | 0 | 9 |  | 0 | 16 |  | 1 | 18 |  | 0 | 16 |  | 1 | 17 |  |

| **Delay (hrs)** | **Tissue** | **Available** | **ALK D5F3** | | | **CK 7 (Dako)** | | | **EGFR (Dako)** | | | **PD-L1 (22c3)** | | | **TTF-1 (Dako)** | | | **Synaptophysin** | | | **Chromogranin** | | | **CD 56** | | |
| --- | --- | --- | --- | --- | --- | --- | --- | --- | --- | --- | --- | --- | --- | --- | --- | --- | --- | --- | --- | --- | --- | --- | --- | --- | --- | --- |
|  |  |  | **No** | **Yes** | **p** | **No** | **Yes** | **p** | **No** | **Yes** | **p** | **No** | **Yes** | **p** | **No** | **Yes** | **p** | **No** | **Yes** | **p** | **No** | **Yes** | **p** | **No** | **Yes** | **p** |
| 1 | normal | No | 0 | 0 | - | 7 | 5 | 1.00 | 9 | 2 | 0.45 |  |  |  | 2 | 2 | 0.50 | 0 | 0 | - | 0 | 5 | 0.22 | 0 | 4 | 0.38 |
|  |  | Yes | 0 | 20 |  | 4 | 4 |  | 5 | 4 |  |  |  |  | 0 | 2 |  | 0 | 20 |  | 1 | 14 |  | 1 | 15 |  |
|  | tumor | No | 0 | 0 | - | 0 | 6 | 0.29 | 0 | 5 | 0.45 | 0 | 4 | 0.69 | 0 | 3 | - | 0 | 0 | - | 0 | 5 | 0.22 | 0 | 4 | 0.38 |
|  |  | Yes | 0 | 20 |  | 2 | 12 |  | 2 | 13 |  | 2 | 14 |  | 0 | 3 |  | 0 | 20 |  | 1 | 14 |  | 1 | 15 |  |
| 6 | normal | No | 0 | 0 | - | 6 | 4 | 1.00 | 12 | 4 | 0.69 |  |  |  | 2 | 2 | 0.50 | 0 | 1 | - | 0 | 3 | 0.63 | 0 | 2 | 1.00 |
|  |  | Yes | 0 | 20 |  | 5 | 5 |  | 2 | 2 |  |  |  |  | 0 | 2 |  | 0 | 19 |  | 1 | 16 |  | 1 | 17 |  |
|  | tumor | No | 0 | 0 | - | 0 | 4 | 0.69 | 0 | 4 | 0.69 | 0 | 4 | 0.69 | 0 | 2 | - | 0 | 1 | - | 0 | 3 | 0.63 | 0 | 2 | 1.00 |
|  |  | Yes | 0 | 20 |  | 2 | 14 |  | 2 | 14 |  | 2 | 14 |  | 0 | 4 |  | 0 | 19 |  | 1 | 16 |  | 1 | 17 |  |
| 24 | normal | No | 0 | 0 | - | 7 | 5 | 1.00 | 12 | 4 | 0.69 |  |  |  | 2 | 3 | 0.25 | 0 | 2 | - | 1 | 2 | 0.50 | 1 | 1 | 1.00 |
|  |  | Yes | 0 | 20 |  | 4 | 4 |  | 2 | 2 |  |  |  |  | 0 | 1 |  | 0 | 18 |  | 0 | 17 |  | 0 | 18 |  |
|  | tumor | No | 0 | 0 | - | 2 | 5 | 0.063 | 2 | 5 | 0.063 | 2 | 6 | **0.031** | 0 | 4 | - | 0 | 2 | - | 1 | 2 | 0.50 | 1 | 1 | 1.00 |
|  |  | Yes | 0 | 20 |  | 0 | 13 |  | 0 | 13 |  | 0 | 12 |  | 0 | 2 |  | 0 | 18 |  | 0 | 17 |  | 0 | 18 |  |
| 48 | normal | No | 0 | 0 | - | 7 | 4 | 1.00 | 12 | 3 | 1.00 |  |  |  | 1 | 1 | 1.00 | 0 | 0 | - | 0 | 1 | 1.00 | 0 | 2 | 1.00 |
|  |  | Yes | 0 | 20 |  | 4 | 5 |  | 2 | 3 |  |  |  |  | 1 | 3 |  | 0 | 20 |  | 1 | 18 |  | 1 | 17 |  |
|  | tumor | No | 0 | 0 | - | 1 | 4 | 0.38 | 1 | 6 | 0.13 | 1 | 4 | 0.38 | 0 | 2 | - | 0 | 0 | - | 0 | 1 | 1.00 | 0 | 2 | 1.00 |
|  |  | Yes | 0 | 20 |  | 1 | 14 |  | 1 | 12 |  | 1 | 14 |  | 0 | 4 |  | 0 | 20 |  | 1 | 18 |  | 1 | 17 |  |
| 96 | normal | No | 0 | 0 | - | 8 | 5 | 0.73 | 11 | 5 | 0.73 |  |  |  | 2 | 4 | - | 0 | 4 | - | 1 | 7 | **0.016** | 0 | 7 | 0.070 |
|  |  | Yes | 0 | 20 |  | 3 | 4 |  | 3 | 1 |  |  |  |  | 0 | 0 |  | 0 | 16 |  | 0 | 12 |  | 1 | 12 |  |
|  | tumor | No | 0 | 0 | - | 1 | 10 | **0.012** | 1 | 5 | 0.22 | 1 | 6 | 0.13 | 0 | 3 | - | 0 | 4 | - | 1 | 7 | **0.016** | 0 | 7 | 0.070 |
|  |  | Yes | 0 | 20 |  | 1 | 8 |  | 1 | 13 |  | 1 | 12 |  | 0 | 3 |  | 0 | 16 |  | 0 | 12 |  | 1 | 12 |  |

| **Prolonged (days)** | **Tissue** | **Available** | **p80** | | | **CK 7 (Monosan)** | | | **Ker MNF 116** | | | **AE13** | | | **Cam 5.2** | | | **TTF-1** | | | **BRAFV600E** | | | **p40** | | |
| --- | --- | --- | --- | --- | --- | --- | --- | --- | --- | --- | --- | --- | --- | --- | --- | --- | --- | --- | --- | --- | --- | --- | --- | --- | --- | --- |
|  |  |  | **No** | **Yes** | **p** | **No** | **Yes** | **p** | **No** | **Yes** | **p** | **No** | **Yes** | **p** | **No** | **Yes** | **p** | **No** | **Yes** | **p** | **No** | **Yes** | **p** | **No** | **Yes** | **p** |
| 2 | normal | No | 5 | 1 | **0.021** | 2 | 3 | 0.23 | 7 | 3 | 0.73 | 6 | 3 | 0.73 | 7 | 2 | 0.45 | 5 | 4 | 1.00 | 11 | 2 | 0.69 | 11 | 3 | 0.73 |
|  |  | Yes | 9 | 5 |  | 8 | 7 |  | 5 | 5 |  | 5 | 6 |  | 5 | 6 |  | 5 | 6 |  | 4 | 3 |  | 5 | 1 |  |
|  | tumor | No | 0 | 4 | 1.00 | 2 | 1 | 0.38 | 1 | 2 | 0.69 | 2 | 4 | 1.00 | 1 | 3 | 1.00 | 1 | 3 | 1.00 | 3 | 1 | 0.63 | 2 | 2 | 1.00 |
|  |  | Yes | 3 | 13 |  | 4 | 13 |  | 4 | 13 |  | 3 | 11 |  | 3 | 13 |  | 2 | 14 |  | 3 | 13 |  | 3 | 13 |  |
| 4 | normal | No | 9 | 2 | 0.45 | 3 | 0 | **0.016** | 8 | 1 | 0.38 | 8 | 4 | 1.00 | 7 | 1 | 0.22 | 8 | 3 | 1.00 | 11 | 2 | 0.69 | 12 | 2 | 0.69 |
|  |  | Yes | 5 | 4 |  | 7 | 10 |  | 4 | 7 |  | 3 | 5 |  | 5 | 7 |  | 2 | 7 |  | 4 | 3 |  | 4 | 2 |  |
|  | tumor | No | 0 | 3 | 1.00 | 1 | 2 | 0.45 | 1 | 3 | 1.00 | 4 | 5 | 0.22 | 1 | 3 | 1.00 | 1 | 3 | 1.00 | 1 | 4 | 1.00 | 2 | 3 | 1.00 |
|  |  | Yes | 3 | 14 |  | 5 | 12 |  | 4 | 12 |  | 1 | 10 |  | 3 | 13 |  | 2 | 14 |  | 5 | 10 |  | 3 | 12 |  |
| 7 | normal | No | 9 | 2 | 0.45 | 0 | 5 | 0.30 | 9 | 2 | 1.00 | 6 | 3 | 0.73 | 7 | 1 | 0.22 | 6 | 4 | 1.00 | 14 | 2 | 1.00 | 14 | 0 | 0.50 |
|  |  | Yes | 5 | 4 |  | 10 | 5 |  | 3 | 6 |  | 5 | 6 |  | 5 | 7 |  | 4 | 6 |  | 1 | 3 |  | 2 | 4 |  |
|  | tumor | No | 1 | 3 | 1.00 | 0 | 1 | 0.13 | 1 | 2 | 0.69 | 3 | 1 | 1.00 | 1 | 3 | 1.00 | 0 | 2 | 1.00 | 0 | 2 | 0.29 | 2 | 3 | 1.00 |
|  |  | Yes | 2 | 14 |  | 6 | 13 |  | 4 | 13 |  | 2 | 14 |  | 3 | 13 |  | 3 | 15 |  | 6 | 12 |  | 3 | 12 |  |

| **Prolonged (days)** | **Tissue** | **Available** | **PD-L1** | | | **ROS1** | | | **C-MET** | | | **p63** | | | **CK 5/6** | | | **Napsin A** | | | **D2-40** | | | **TTF-1 (Ventana)** | | |
| --- | --- | --- | --- | --- | --- | --- | --- | --- | --- | --- | --- | --- | --- | --- | --- | --- | --- | --- | --- | --- | --- | --- | --- | --- | --- | --- |
|  |  |  | **No** | **Yes** | **p** | **No** | **Yes** | **p** | **No** | **Yes** | **p** | **No** | **Yes** | **p** | **No** | **Yes** | **p** | **No** | **Yes** | **p** | **No** | **Yes** | **p** | **No** | **Yes** | **p** |
| 2 | normal | No | 1 | 2 | 1.00 | 5 | 5 | 0.77 | 9 | 2 | 0.45 | 13 | 3 | 1.00 | 0 | 0 | - | 0 | 0 | - | 0 | 0 | - | 4 | 5 | 1.00 |
|  |  | Yes | 3 | 14 |  | 7 | 3 |  | 5 | 4 |  | 2 | 2 |  | 0 | 20 |  | 1 | 19 |  | 0 | 20 |  | 6 | 5 |  |
|  | tumor | No | 0 | 4 | 1.00 | 1 | 4 | 1.00 | 2 | 3 | 1.00 | 1 | 4 | 0.69 | 0 | 0 | - | 0 | 1 | 1.00 | 0 | 0 | - | 0 | 3 | 1.00 |
|  |  | Yes | 5 | 11 |  | 4 | 11 |  | 3 | 12 |  | 2 | 13 |  | 1 | 19 |  | 2 | 17 |  | 1 | 19 |  | 3 | 14 |  |
| 4 | normal | No | 1 | 4 | 1.00 | 9 | 3 | 1.00 | 10 | 1 | 0.38 | 15 | 4 | 0.13 | 0 | 0 | - | 0 | 1 | 1.00 | 0 | 0 | - | 5 | 3 | 0.73 |
|  |  | Yes | 3 | 12 |  | 3 | 5 |  | 4 | 5 |  | 0 | 1 |  | 0 | 20 |  | 1 | 18 |  | 0 | 20 |  | 5 | 7 |  |
|  | tumor | No | 1 | 5 | 1.00 | 0 | 1 | 0.22 | 1 | 4 | 1.00 | 1 | 4 | 0.69 | 0 | 0 | - | 1 | 1 | 1.00 | 0 | 1 | 1.00 | 0 | 2 | 1.00 |
|  |  | Yes | 4 | 10 |  | 5 | 14 |  | 4 | 11 |  | 2 | 13 |  | 1 | 19 |  | 1 | 17 |  | 1 | 18 |  | 3 | 15 |  |
| 7 | normal | No | 1 | 1 | 0.63 | 6 | 2 | 0.29 | 12 | 4 | 0.69 | 14 | 5 | 0.22 | 0 | 1 | - | 0 | 0 | - | 0 | 1 | - | 5 | 1 | 0.22 |
|  |  | Yes | 3 | 15 |  | 6 | 6 |  | 2 | 2 |  | 1 | 0 |  | 0 | 19 |  | 1 | 19 |  | 0 | 19 |  | 5 | 9 |  |
|  | tumor | No | 2 | 2 | 1.00 | 0 | 1 | 0.22 | 2 | 3 | 1.00 | 1 | 2 | 1.00 | 0 | 1 | 1.00 | 0 | 4 | 0.69 | 0 | 1 | 1.00 | 0 | 3 | 1.00 |
|  |  | Yes | 3 | 13 |  | 5 | 14 |  | 3 | 12 |  | 2 | 15 |  | 1 | 18 |  | 2 | 14 |  | 1 | 18 |  | 3 | 14 |  |

| **Prolonged (days)** | **Tissue** | **Available** | **ALK D5F3** | | | **CK 7 (Dako)** | | | **EGFR (Dako)** | | | **PD-L1 (22c3)** | | | **TTF-1 (Dako)** | | | **Synaptophysin** | | | **Chromogranin** | | | **CD 56** | | |
| --- | --- | --- | --- | --- | --- | --- | --- | --- | --- | --- | --- | --- | --- | --- | --- | --- | --- | --- | --- | --- | --- | --- | --- | --- | --- | --- |
|  |  |  | **No** | **Yes** | **p** | **No** | **Yes** | **p** | **No** | **Yes** | **p** | **No** | **Yes** | **p** | **No** | **Yes** | **p** | **No** | **Yes** | **p** | **No** | **Yes** | **p** | **No** | **Yes** | **p** |
| 2 | normal | No | 0 | 0 | - | 5 | 5 | 1.00 | 7 | 2 | 0.45 |  |  |  | 3 | 3 | 1.00 | 0 | 0 | - | 0 | 3 | 1.00 | 0 | 2 | 1.00 |
|  |  | Yes | 0 | 20 |  | 4 | 6 |  | 5 | 6 |  |  |  |  | 4 | 4 |  | 1 | 19 |  | 4 | 13 |  | 3 | 15 |  |
|  | tumor | No | 0 | 0 | - | 0 | 2 | 0.69 | 1 | 3 | 1.00 | 0 | 4 | 1.00 | 4 | 1 | 0.63 | 0 | 0 | - | 0 | 3 | 1.00 | 0 | 2 | 1.00 |
|  |  | Yes | 0 | 20 |  | 4 | 14 |  | 4 | 12 |  | 5 | 11 |  | 3 | 6 |  | 1 | 19 |  | 4 | 13 |  | 3 | 15 |  |
| 4 | normal | No | 0 | 0 | - | 6 | 3 | 1.00 | 9 | 3 | 1.00 |  |  |  | 6 | 3 | 0.63 | 1 | 0 | 1.00 | 1 | 2 | 1.00 | 1 | 1 | 1.00 |
|  |  | Yes | 0 | 20 |  | 3 | 8 |  | 3 | 5 |  |  |  |  | 1 | 4 |  | 0 | 19 |  | 3 | 14 |  | 2 | 16 |  |
|  | tumor | No | 0 | 0 | - | 1 | 2 | 1.00 | 1 | 2 | 0.69 | 1 | 2 | 0.69 | 4 | 0 | 0.25 | 1 | 0 | 1.00 | 1 | 2 | 1.00 | 1 | 1 | 1.00 |
|  |  | Yes | 0 | 20 |  | 3 | 14 |  | 4 | 13 |  | 4 | 13 |  | 3 | 7 |  | 0 | 19 |  | 3 | 14 |  | 2 | 16 |  |
| 7 | normal | No | 0 | 0 | - | 5 | 3 | 1.00 | 9 | 1 | 0.63 |  |  |  | 4 | 2 | 1.00 | 0 | 2 | 1.00 | 1 | 3 | 1.00 | 1 | 2 | 1.00 |
|  |  | Yes | 0 | 20 |  | 4 | 8 |  | 3 | 7 |  |  |  |  | 3 | 5 |  | 1 | 17 |  | 3 | 13 |  | 2 | 15 |  |
|  | tumor | No | 0 | 0 | - | 1 | 2 | 1.00 | 2 | 3 | 1.00 | 2 | 3 | 1.00 | 5 | 1 | 1.00 | 0 | 2 | 1.00 | 1 | 3 | 1.00 | 1 | 2 | 1.00 |
|  |  | Yes | 0 | 20 |  | 3 | 14 |  | 3 | 12 |  | 3 | 12 |  | 2 | 6 |  | 1 | 17 |  | 3 | 13 |  | 2 | 15 |  |
