# Supplementary material for: Impact of delayed and prolonged fixation on the evaluation of immunohistochemical staining on lung carcinoma resection specimen
Source: Virchows Arch. 2019 Jul 1;475(2):191–9. doi: 10.1007/s00428-019-02595-9 (PMC6647403; doi:10.1007/s00428-019-02595-9)
Supplement: Supplementary file 3 — (DOCX 112 kb) [file 428_2019_2595_MOESM3_ESM.docx]

Supplementary table 3 *Number of cores with poor quality (score 5) for standard versus delayed/prolonged fixation for each antibody*

| **Delay (hrs)** | **Tissue** | **Score** | **p80** | | | **CK7 (Monosan)** | | | **Ker MNF 116** | | | **AE13** | | | **CAM 5.2** | | | **TTF-1 (Dako)** | | | **BRAFV600E** | | | **p40** | | |
| --- | --- | --- | --- | --- | --- | --- | --- | --- | --- | --- | --- | --- | --- | --- | --- | --- | --- | --- | --- | --- | --- | --- | --- | --- | --- | --- |
|  |  |  | **1-4** | **5** | **p-value** | **1-4** | **5** | **p-value** | **1-4** | **5** | **p-value** | **1-4** | **5** | **p-value** | **1-4** | **5** | **p-value** | **1-4** | **5** | **p-value** | **1-4** | **5** | **p-value** | **1-4** | **5** | **p-value** |
| 1 | normal | Intensity IHC | 2 | 0 | - | 12 | 1 | 1.00 | 6 | 0 | - | 4 | 0 | - | 6 | 0 | - | 6 | 0 | - | 2 | 0 | - | 3 | 0 | - |
|  |  | Poor Quality | 0 | 0 |  | 1 | 0 |  | 0 | 0 |  | 1 | 0 |  | 0 | 0 |  | 0 | 0 |  | 0 | 0 |  | 0 | 0 |  |
|  | tumor | Intensity IHC | 12 | 0 | - | 12 | 1 | 1.00 | 14 | 1 | 0.63 | 12 | 1 | 0.22 | 13 | 0 | - | 12 | 0 | - | 13 | 0 | - | 13 | 0 | - |
|  |  | Poor Quality | 0 | 0 |  | 1 | 0 |  | 3 | 0 |  | 5 | 0 |  | 2 | 0 |  | 1 | 0 |  | 0 | 0 |  | 0 | 0 |  |
| 6 | normal | Intensity IHC | 3 | 0 | - | 12 | 1 | 1.00 | 4 | 0 | - | 4 | 0 | - | 3 | 0 | - | 3 | 0 | - | 1 | 0 | - | 3 | 0 | - |
|  |  | Poor Quality | 0 | 0 |  | 1 | 0 |  | 0 | 0 |  | 0 | 0 |  | 0 | 0 |  | 0 | 0 |  | 0 | 0 |  | 0 | 0 |  |
|  | tumor | Intensity IHC | 16 | 0 | - | 16 | 1 | - | 16 | 1 | - | 14 | 1 | 1.00 | 15 | 0 | - | 15 | 0 | - | 16 | 0 | - | 15 | 0 | - |
|  |  | Poor Quality | 0 | 0 |  | 0 | 0 |  | 0 | 0 |  | 2 | 0 |  | 0 | 0 |  | 0 | 0 |  | 0 | 0 |  | 0 | 0 |  |
| 24 | normal | Intensity IHC | 4 | 0 | - | 4 | 0 | - | 4 | 0 | - | 3 | 0 | 1.00 | 2 | 0 | - | 3 | 0 | - | 1 | 0 | - | 3 | 0 | - |
|  |  | Poor Quality | 1 | 0 |  | 2 | 0 |  | 3 | 0 |  | 1 | 1 |  | 4 | 0 |  | 2 | 0 |  | 1 | 0 |  | 0 | 0 |  |
|  | tumor | Intensity IHC | 13 | 0 | - | 9 | 0 | - | 11 | 0 | - | 13 | 0 | 0.50 | 9 | 0 | - | 12 | 0 | - | 12 | 0 | - | 11 | 0 | - |
|  |  | Poor Quality | 3 | 0 |  | 1 | 0 |  | 3 | 0 |  | 2 | 1 |  | 4 | 0 |  | 2 | 0 |  | 1 | 0 |  | 0 | 0 |  |
| 48 | normal | Intensity IHC | 2 | 0 | - | 8 | 1 | - | 4 | 1 | - | 2 | 0 | 1.00 | 4 | 0 | - | 3 | 0 | - | 1 | 0 | - | 3 | 0 | - |
|  |  | Poor Quality | 3 | 0 |  | 0 | 0 |  | 0 | 0 |  | 0 | 1 |  | 0 | 0 |  | 0 | 0 |  | 1 | 0 |  | 0 | 0 |  |
|  | tumor | Intensity IHC | 12 | 0 | - | 8 | 1 | 1.00 | 14 | 1 | 1.00 | 14 | 1 | 0.63 | 8 | 0 | - | 12 | 0 | - | 14 | 0 | - | 9 | 0 | - |
|  |  | Poor Quality | 3 | 0 |  | 1 | 0 |  | 1 | 0 |  | 3 | 0 |  | 1 | 0 |  | 2 | 0 |  | 1 | 0 |  | 1 | 0 |  |
| 96 | normal | Intensity IHC | 1 | 0 | - | 2 | 0 | - | 1 | 0 | **0.031** | 0 | 0 | - | 1 | 0 | - | 0 | 0 | - | 0 | 0 | - | 0 | 0 | - |
|  |  | Poor Quality | 4 | 0 |  | 6 | 0 |  | 6 | 1 |  | 2 | 1 |  | 2 | 0 |  | 0 | 0 |  | 3 | 0 |  | 0 | 0 |  |
|  | tumor | Intensity IHC | 10 | 0 | - | 6 | 0 | - | 11 | 0 | - | 7 | 0 | **0.008** | 6 | 0 | - | 8 | 0 | - | 10 | 0 | - | 4 | 0 | - |
|  |  | Poor Quality | 4 | 0 |  | 4 | 0 |  | 6 | 0 |  | 8 | 1 |  | 3 | 0 |  | 2 | 0 |  | 5 | 0 |  | 1 | 0 |  |

| **Delay (hrs)** | **Tissue** | **Score** | **PD-L1** | | | **ROS1** | | | **C-MET** | | | **p63** | | | **CK 5/6** | | | **Napsin A** | | | **D2-40** | | | **TTF-1 (Ventana)** | | |
| --- | --- | --- | --- | --- | --- | --- | --- | --- | --- | --- | --- | --- | --- | --- | --- | --- | --- | --- | --- | --- | --- | --- | --- | --- | --- | --- |
|  |  |  | **1-4** | **5** | **p-value** | **1-4** | **5** | **p-value** | **1-4** | **5** | **p-value** | **1-4** | **5** | **p-value** | **1-4** | **5** | **p-value** | **1-4** | **5** | **p-value** | **1-4** | **5** | **p-value** | **1-4** | **5** | **p-value** |
| 1 | normal | Intensity IHC | 16 | 0 | - | 3 | 0 | - | 5 | 0 | - | 0 | 0 | - | 20 | 0 | - | 20 | 0 | - | 20 | 0 | - | 6 | 0 | - |
|  |  | Poor Quality | 0 | 0 |  | 0 | 0 |  | 0 | 0 |  | 0 | 0 |  | 0 | 0 |  | 0 | 0 |  | 0 | 0 |  | 0 | 0 |  |
|  | tumor | Intensity IHC | 12 | 0 | - | 11 | 0 | - | 13 | 0 | - | 12 | 0 | - | 20 | 0 | - | 18 | 0 | - | 18 | 0 | - | 13 | 0 | - |
|  |  | Poor Quality | 0 | 0 |  | 1 | 0 |  | 0 | 0 |  | 1 | 0 |  | 0 | 0 |  | 0 | 0 |  | 0 | 0 |  | 1 | 0 |  |
| 6 | normal | Intensity IHC | 17 | 0 | - | 3 | 0 | - | 6 | 0 | - | 0 | 0 | - | 19 | 0 | - | 19 | 0 | - | 19 | 0 | - | 6 | 0 | - |
|  |  | Poor Quality | 0 | 0 |  | 0 | 0 |  | 0 | 0 |  | 0 | 0 |  | 0 | 0 |  | 0 | 0 |  | 0 | 0 |  | 0 | 0 |  |
|  | tumor | Intensity IHC | 15 | 0 | - | 16 | 0 | - | 16 | 0 | - | 15 | 0 | - | 19 | 0 | - | 17 | 0 | - | 18 | 0 | - | 16 | 0 | - |
|  |  | Poor Quality | 0 | 0 |  | 0 | 0 |  | 0 | 0 |  | 1 | 0 |  | 0 | 0 |  | 0 | 0 |  | 0 | 0 |  | 0 | 0 |  |
| 24 | normal | Intensity IHC | 14 | 0 | - | 2 | 0 | - | 3 | 0 | - | 0 | 0 | - | 19 | 0 | - | 20 | 0 | - | 20 | 0 | - | 4 | 0 | - |
|  |  | Poor Quality | 1 | 0 |  | 2 | 0 |  | 0 | 0 |  | 0 | 0 |  | 0 | 0 |  | 0 | 0 |  | 0 | 0 |  | 1 | 0 |  |
|  | tumor | Intensity IHC | 10 | 0 | - | 15 | 0 | - | 12 | 0 | - | 13 | 0 | - | 17 | 0 | - | 18 | 0 | - | 18 | 0 | - | 12 | 0 | - |
|  |  | Poor Quality | 1 | 0 |  | 1 | 0 |  | 0 | 0 |  | 3 | 0 |  | 0 | 0 |  | 0 | 0 |  | 0 | 0 |  | 1 | 0 |  |
| 48 | normal | Intensity IHC | 13 | 0 | - | 3 | 0 | - | 3 | 0 | - | 0 | 0 | - | 18 | 0 | - | 20 | 0 | - | 19 | 0 | - | 4 | 0 | - |
|  |  | Poor Quality | 2 | 0 |  | 2 | 0 |  | 0 | 0 |  | 0 | 0 |  | 0 | 0 |  | 0 | 0 |  | 0 | 0 |  | 0 | 0 |  |
|  | tumor | Intensity IHC | 11 | 0 | - | 13 | 0 | - | 10 | 0 | - | 11 | 0 | - | 18 | 0 | - | 19 | 0 | - | 17 | 0 | - | 13 | 0 | - |
|  |  | Poor Quality | 2 | 0 |  | 0 | 0 |  | 0 | 0 |  | 3 | 0 |  | 0 | 0 |  | 0 | 0 |  | 0 | 0 |  | 2 | 0 |  |
| 96 | normal | Intensity IHC | 11 | 1 | 0.22 | 2 | 0 | - | 1 | 0 | - | 0 | 0 | - | 16 | 0 | - | 20 | 0 | - | 15 | 0 | - | 4 | 0 | - |
|  |  | Poor Quality | 5 | 0 |  | 3 | 0 |  | 0 | 0 |  | 0 | 0 |  | 0 | 0 |  | 0 | 0 |  | 0 | 0 |  | 1 | 0 |  |
|  | tumor | Intensity IHC | 9 | 1 | 0.22 | 10 | 0 | - | 11 | 0 | - | 6 | 0 | - | 16 | 0 | - | 18 | 0 | - | 16 | 0 | - | 15 | 0 | - |
|  |  | Poor Quality | 5 | 0 |  | 3 | 0 |  | 0 | 0 |  | 3 | 0 |  | 0 | 0 |  | 0 | 0 |  | 0 | 0 |  | 2 | 0 |  |

| **Delay (hrs)** | **Tissue** | **Score** | **ALK D5F3** | | | **CK 7 (Dako)** | | | **EGFR (Dako)** | | | **PD-L1 (22c3)** | | | **TTF-1 (Dako)** | | | **Synaptophysin** | | | **Chromogranin** | | | **CD 56** | | |
| --- | --- | --- | --- | --- | --- | --- | --- | --- | --- | --- | --- | --- | --- | --- | --- | --- | --- | --- | --- | --- | --- | --- | --- | --- | --- | --- |
|  |  |  | **1-4** | **5** | **p-value** | **1-4** | **5** | **p-value** | **1-4** | **5** | **p-value** | **1-4** | **5** | **p-value** | **1-4** | **5** | **p-value** | **1-4** | **5** | **p-value** | **1-4** | **5** | **p-value** | **1-4** | **5** | **p-value** |
| 1 | normal | Intensity IHC | 20 | 0 | - | 4 | 0 | - | 4 | 0 | - | 0 | 0 | - | 1 | 1 | - | 7 | 1 | **0.006** | 8 | 1 | 0.38 | 9 | 3 | 1.00 |
|  |  | Poor Quality | 0 | 0 |  | 0 | 0 |  | 0 | 0 |  | 0 | 0 |  | 0 | 0 |  | 11 | 1 |  | 4 | 1 |  | 2 | 1 |  |
|  | tumor | Intensity IHC | 20 | 0 | - | 12 | 0 | - | 12 | 0 | - | 14 | 0 | - | 2 | 1 | - | 7 | 1 | **0.006** | 8 | 1 | 0.38 | 9 | 3 | 1.00 |
|  |  | Poor Quality | 0 | 0 |  | 0 | 0 |  | 1 | 0 |  | 0 | 0 |  | 0 | 0 |  | 11 | 1 |  | 4 | 1 |  | 2 | 1 |  |
| 6 | normal | Intensity IHC | 20 | 0 | - | 5 | 0 | - | 2 | 0 | - | 0 | 0 | - | 2 | 0 | - | 13 | 1 | 0.38 | 12 | 2 | 1.00 | 10 | 3 | 1.00 |
|  |  | Poor Quality | 0 | 0 |  | 0 | 0 |  | 0 | 0 |  | 0 | 0 |  | 0 | 0 |  | 4 | 1 |  | 2 | 0 |  | 3 | 1 |  |
|  | tumor | Intensity IHC | 20 | 0 | - | 13 | 0 | - | 14 | 0 | - | 14 | 0 | - | 3 | 1 | - | 13 | 1 | 0.38 | 12 | 2 | 1.00 | 10 | 3 | 1.00 |
|  |  | Poor Quality | 0 | 0 |  | 1 | 0 |  | 0 | 0 |  | 0 | 0 |  | 0 | 0 |  | 4 | 1 |  | 2 | 0 |  | 3 | 1 |  |
| 24 | normal | Intensity IHC | 20 | 0 | - | 3 | 0 | - | 0 | 0 | - | 0 | 0 | - | 1 | 0 | - | 9 | 0 | **0.008** | 12 | 0 | 0.25 | 6 | 2 | 0.11 |
|  |  | Poor Quality | 0 | 0 |  | 1 | 0 |  | 2 | 0 |  | 0 | 0 |  | 0 | 0 |  | 8 | 1 |  | 3 | 2 |  | 8 | 2 |  |
|  | tumor | Intensity IHC | 20 | 0 | - | 11 | 0 | - | 11 | 0 | - | 10 | 0 | - | 2 | 0 | - | 9 | 0 | **0.008** | 11 | 0 | 0.13 | 6 | 2 | 0.11 |
|  |  | Poor Quality | 0 | 0 |  | 2 | 0 |  | 2 | 0 |  | 2 | 0 |  | 0 | 0 |  | 8 | 1 |  | 4 | 2 |  | 8 | 2 |  |
| 48 | normal | Intensity IHC | 20 | 0 | - | 5 | 0 | - | 3 | 0 | - | 0 | 0 | - | 2 | 1 | - | 10 | 1 | **0.039** | 15 | 1 | 1.00 | 11 | 2 | 1.00 |
|  |  | Poor Quality | 0 | 0 |  | 0 | 0 |  | 0 | 0 |  | 0 | 0 |  | 0 | 0 |  | 8 | 1 |  | 1 | 1 |  | 2 | 2 |  |
|  | tumor | Intensity IHC | 20 | 0 | - | 13 | 0 | - | 12 | 0 | - | 14 | 0 | - | 3 | 1 | - | 10 | 1 | **0.039** | 15 | 1 | 1.00 | 11 | 2 | 1.00 |
|  |  | Poor Quality | 0 | 0 |  | 1 | 0 |  | 0 | 0 |  | 0 | 0 |  | 0 | 0 |  | 8 | 1 |  | 1 | 1 |  | 2 | 2 |  |
| 96 | normal | Intensity IHC | 20 | 0 | - | 4 | 0 | - | 0 | 0 | - | 0 | 0 | - | 0 | 0 | - | 7 | 0 | - | 7 | 0 | 0.13 | 4 | 0 | **0.031** |
|  |  | Poor Quality | 0 | 0 |  | 0 | 0 |  | 1 | 0 |  | 0 | 0 |  | 0 | 0 |  | 9 | 0 |  | 4 | 1 |  | 6 | 2 |  |
|  | tumor | Intensity IHC | 20 | 0 | - | 8 | 0 | - | 11 | 0 | - | 12 | 0 | - | 2 | 0 | - | 7 | 0 | - | 6 | 0 | 0.06 | 4 | 0 | **0.031** |
|  |  | Poor Quality | 0 | 0 |  | 0 | 0 |  | 2 | 0 |  | 0 | 0 |  | 1 | 0 |  | 9 | 0 |  | 5 | 1 |  | 6 | 2 |  |

| **Prolonged (days)** | **Tissue** | **Score** | **p80** | | | **CK 7 (Monosan)** | | | **Ker MNF 116** | | | **AE13** | | | **CAM 5.2** | | | **TTF-1 (Dako)** | | | **BRAFV600E** | | | **p40** | | |
| --- | --- | --- | --- | --- | --- | --- | --- | --- | --- | --- | --- | --- | --- | --- | --- | --- | --- | --- | --- | --- | --- | --- | --- | --- | --- | --- |
|  |  |  | **1-4** | **5** | **p-value** | **1-4** | **5** | **p-value** | **1-4** | **5** | **p-value** | **1-4** | **5** | **p-value** | **1-4** | **5** | **p-value** | **1-4** | **5** | **p-value** | **1-4** | **5** | **p-value** | **1-4** | **5** | **p-value** |
| 2 | normal | Intensity IHC | 5 | 0 | - | 4 | 2 | 1.00 | 5 | 0 | - | 3 | 1 | 1.00 | 6 | 0 | - | 6 | 0 | - | 3 | 0 | - | 1 | 0 | - |
|  |  | Poor Quality | 0 | 0 |  | 1 | 0 |  | 0 | 0 |  | 1 | 1 |  | 0 | 0 |  | 0 | 0 |  | 0 | 0 |  | 0 | 0 |  |
|  | tumor | Intensity IHC | 13 | 0 | - | 11 | 1 | 1.00 | 13 | 0 | - | 8 | 1 | 1.00 | 11 | 0 | - | 12 | 1 | 1.00 | 13 | 0 | - | 13 | 0 | - |
|  |  | Poor Quality | 0 | 0 |  | 1 | 0 |  | 0 | 0 |  | 1 | 1 |  | 2 | 0 |  | 1 | 0 |  | 0 | 0 |  | 0 | 0 |  |
| 4 | normal | Intensity IHC | 4 | 0 | - | 6 | 1 | 1.00 | 7 | 0 | - | 4 | 0 | 1.00 | 7 | 0 | - | 7 | 0 | - | 3 | 0 | - | 2 | 0 | - |
|  |  | Poor Quality | 0 | 0 |  | 1 | 2 |  | 0 | 0 |  | 0 | 1 |  | 0 | 0 |  | 0 | 0 |  | 0 | 0 |  | 0 | 0 |  |
|  | tumor | Intensity IHC | 14 | 0 | - | 10 | 1 | 1.00 | 11 | 0 | - | 8 | 1 | 1.00 | 11 | 0 | - | 13 | 1 | - | 10 | 0 | - | 12 | 0 | - |
|  |  | Poor Quality | 0 | 0 |  | 1 | 0 |  | 1 | 0 |  | 0 | 1 |  | 2 | 0 |  | 0 | 0 |  | 0 | 0 |  | 0 | 0 |  |
| 7 | normal | Intensity IHC | 4 | 0 | - | 4 | 1 | - | 6 | 0 | - | 4 | 0 | 1.00 | 7 | 0 | - | 6 | 0 | - | 3 | 0 | - | 4 | 0 | - |
|  |  | Poor Quality | 0 | 0 |  | 0 | 0 |  | 0 | 0 |  | 1 | 1 |  | 0 | 0 |  | 0 | 0 |  | 0 | 0 |  | 0 | 0 |  |
|  | tumor | Intensity IHC | 14 | 0 | - | 11 | 1 | 1.00 | 12 | 0 | - | 10 | 2 | 1.00 | 11 | 0 | - | 14 | 1 | - | 12 | 0 | - | 12 | 0 | - |
|  |  | Poor Quality | 0 | 0 |  | 1 | 0 |  | 1 | 0 |  | 2 | 0 |  | 2 | 0 |  | 0 | 0 |  | 0 | 0 |  | 0 | 0 |  |

| **Prolonged (days)** | **Tissue** | **Score** | **PD-L1** | | | **ROS1** | | | **C-MET** | | | **p63** | | | **CK 5/6** | | | **Napsin A** | | | **D2-40** | | | **TTF-1 (Ventana)** | | |
| --- | --- | --- | --- | --- | --- | --- | --- | --- | --- | --- | --- | --- | --- | --- | --- | --- | --- | --- | --- | --- | --- | --- | --- | --- | --- | --- |
|  |  |  | **1-4** | **5** | **p-value** | **1-4** | **5** | **p-value** | **1-4** | **5** | **p-value** | **1-4** | **5** | **p-value** | **1-4** | **5** | **p-value** | **1-4** | **5** | **p-value** | **1-4** | **5** | **p-value** | **1-4** | **5** | **p-value** |
| 2 | normal | Intensity IHC | 14 | 0 | - | 2 | 0 | - | 4 | 0 | - | 1 | 1 | - | 20 | 0 | - | 19 | 0 | - | 20 | 0 | - | 5 | 0 | - |
|  |  | Poor Quality | 0 | 0 |  | 1 | 0 |  | 0 | 0 |  | 0 | 0 |  | 0 | 0 |  | 0 | 0 |  | 0 | 0 |  | 0 | 0 |  |
|  | tumor | Intensity IHC | 11 | 0 | - | 10 | 0 | - | 12 | 0 | - | 11 | 2 | - | 19 | 0 | - | 17 | 0 | - | 19 | 0 | - | 14 | 0 | - |
|  |  | Poor Quality | 0 | 0 |  | 1 | 0 |  | 0 | 0 |  | 0 | 0 |  | 0 | 0 |  | 0 | 0 |  | 0 | 0 |  | 0 | 0 |  |
| 4 | normal | Intensity IHC | 11 | 1 | - | 5 | 0 | - | 5 | 0 | - | 1 | 0 | - | 20 | 0 | - | 18 | 0 | - | 20 | 0 | - | 7 | 0 | - |
|  |  | Poor Quality | 0 | 0 |  | 0 | 0 |  | 0 | 0 |  | 0 | 0 |  | 0 | 0 |  | 0 | 0 |  | 0 | 0 |  | 0 | 0 |  |
|  | tumor | Intensity IHC | 9 | 1 | - | 14 | 0 | - | 11 | 0 | - | 11 | 2 | - | 19 | 0 | - | 17 | 0 | - | 18 | 0 | - | 15 | 0 | - |
|  |  | Poor Quality | 0 | 0 |  | 0 | 0 |  | 0 | 0 |  | 0 | 0 |  | 0 | 0 |  | 0 | 0 |  | 0 | 0 |  | 0 | 0 |  |
| 7 | normal | Intensity IHC | 14 | 1 | - | 5 | 0 | - | 2 | 0 | - | 0 | 0 | - | 19 | 0 | - | 19 | 0 | - | 19 | 0 | - | 9 | 0 | - |
|  |  | Poor Quality | 0 | 0 |  | 1 | 0 |  | 0 | 0 |  | 0 | 0 |  | 0 | 0 |  | 0 | 0 |  | 0 | 0 |  | 0 | 0 |  |
|  | tumor | Intensity IHC | 12 | 1 | - | 14 | 0 | - | 12 | 0 | - | 12 | 3 | - | 18 | 0 | - | 14 | 0 | - | 18 | 0 | - | 14 | 0 | - |
|  |  | Poor Quality | 0 | 0 |  | 0 | 0 |  | 0 | 0 |  | 0 | 0 |  | 0 | 0 |  | 0 | 0 |  | 0 | 0 |  | 0 | 0 |  |

| **Prolonged (days)** | **Tissue** | **Score** | **ALK D5F3** | | | **CK 7** | | | **EGFR (Dako)** | | | **PD-L1 (22c3)** | | | **TTF-1 (Dako)** | | | **Synaptophysin** | | | **Chromogranin** | | | **CD 56** | | |
| --- | --- | --- | --- | --- | --- | --- | --- | --- | --- | --- | --- | --- | --- | --- | --- | --- | --- | --- | --- | --- | --- | --- | --- | --- | --- | --- |
|  |  |  | **1-4** | **5** | **p-value** | **1-4** | **5** | **p-value** | **1-4** | **5** | **p-value** | **1-4** | **5** | **p-value** | **1-4** | **5** | **p-value** | **1-4** | **5** | **p-value** | **1-4** | **5** | **p-value** | **1-4** | **5** | **p-value** |
| 2 | normal | Intensity IHC | 20 | 0 | - | 6 | 0 | - | 6 | 0 | - | 0 | 0 | - | 4 | 0 | - | 9 | 5 | 1.00 | 10 | 1 | 1.00 | 10 | 2 | 1.00 |
|  |  | Poor Quality | 0 | 0 |  | 0 | 0 |  | 0 | 0 |  | 0 | 0 |  | 0 | 0 |  | 5 | 0 |  | 1 | 1 |  | 2 | 1 |  |
|  | tumor | Intensity IHC | 20 | 0 | - | 14 | 0 | - | 12 | 0 | - | 11 | 0 | - | 6 | 0 | - | 9 | 5 | 1.00 | 10 | 1 | 1.00 | 10 | 2 | 1.00 |
|  |  | Poor Quality | 0 | 0 |  | 0 | 0 |  | 0 | 0 |  | 0 | 0 |  | 0 | 0 |  | 5 | 0 |  | 1 | 1 |  | 2 | 1 |  |
| 4 | normal | Intensity IHC | 20 | 0 | - | 8 | 0 | - | 5 | 0 | - | 0 | 0 | - | 4 | 0 | - | 11 | 4 | 1.00 | 11 | 2 | 1.00 | 10 | 3 | 1.00 |
|  |  | Poor Quality | 0 | 0 |  | 0 | 0 |  | 0 | 0 |  | 0 | 0 |  | 0 | 0 |  | 3 | 1 |  | 1 | 0 |  | 3 | 0 |  |
|  | tumor | Intensity IHC | 20 | 0 | - | 14 | 0 | - | 13 | 0 | - | 13 | 0 | - | 7 | 0 | - | 11 | 4 | 1.00 | 11 | 2 | 1.00 | 10 | 3 | 1.00 |
|  |  | Poor Quality | 0 | 0 |  | 0 | 0 |  | 0 | 0 |  | 0 | 0 |  | 0 | 0 |  | 3 | 1 |  | 1 | 0 |  | 3 | 0 |  |
| 7 | normal | Intensity IHC | 20 | 0 | - | 8 | 0 | - | 7 | 0 | - | 0 | 0 | - | 5 | 0 | - | 9 | 3 | 0.73 | 11 | 1 | 1.00 | 11 | 1 | 1.00 |
|  |  | Poor Quality | 0 | 0 |  | 0 | 0 |  | 0 | 0 |  | 0 | 0 |  | 0 | 0 |  | 5 | 0 |  | 0 | 1 |  | 2 | 1 |  |
|  | tumor | Intensity IHC | 20 | 0 | - | 14 | 0 | - | 12 | 0 | - | 12 | 0 | - | 6 | 0 | - | 9 | 3 | 0.73 | 11 | 1 | 1.00 | 11 | 1 | 1.00 |
|  |  | Poor Quality | 0 | 0 |  | 0 | 0 |  | 0 | 0 |  | 0 | 0 |  | 0 | 0 |  | 5 | 0 |  | 0 | 1 |  | 2 | 1 |  |

Legend: score 1 negative, 2 weak positive (+), 3 moderate positive (++), 4 strong positive (+++) and 5 poor quality
