# Supplementary material for: Impact of delayed and prolonged fixation on the evaluation of immunohistochemical staining on lung carcinoma resection specimen
Source: Virchows Arch. 2019 Jul 1;475(2):191–9. doi: 10.1007/s00428-019-02595-9 (PMC6647403; doi:10.1007/s00428-019-02595-9)
Supplement: Supplementary file 4 — (DOCX 64 kb) [file 428_2019_2595_MOESM4_ESM.docx]

Supplementary table 4 *For each antibody* *within the number of available cores with an IHC score (ranging from 1 to 4) the p-values comparing staining intensity for delayed and prolonged fixation to standard fixation.*

| **Sample** | **Tissue** | **p80** | | **Keratin 7** | | **Ker MNF116** | | **AE13** | | **Cam 5.2** | | **TTF1 (Dako)** | | **BrafV600E** | | **p40** | |
| --- | --- | --- | --- | --- | --- | --- | --- | --- | --- | --- | --- | --- | --- | --- | --- | --- | --- |
|  |  | **n** | **p-value** | **n** | **p-value** | **n** | **p-value** | **n** | **p-value** | **n** | **p-value** | **n** | **p-value** | **n** | **p-value** | **n** | **p-value** |
| 1h delay | normal | 2 | 1.00 | 12 | 0.18 | 6 | 0.32 | 4 | 1.00 | 6 | 0.56 | 6 | 0.16 | 2 | 1.00 | 3 | 1.00 |
|  | tumor | 12 | 1.00 | 12 | 1.00 | 14 | 0.41 | 12 | 0.32 | 13 | 0.38 | 12 | 0.32 | 13 | 1.00 | 13 | 0.32 |
| 6h delay | normal | 3 | 1.00 | 12 | 0.21 | 4 | 1.00 | 4 | 1.00 | 3 | 1.00 | 3 | 0.16 | 1 | - | 3 | 1.00 |
|  | tumor | 16 | 1.00 | 16 | 0.71 | 16 | 0.56 | 14 | 1.00 | 15 | 1.00 | 15 | 0.10 | 16 | 1.00 | 15 | 1.00 |
| 24h delay | normal | 4 | 1.00 | 4 | 0.083 | 4 | 0.32 | 3 | 1.00 | 2 | 1.00 | 3 | 0.16 | 1 | - | 3 | 1.00 |
|  | tumor | 13 | 1.00 | 9 | 0.32 | 11 | 0.71 | 13 | 0.48 | 9 | 0.71 | 12 | **0.041** | 12 | 1.00 | 11 | 0.79 |
| 48h delay | normal | 2 | 1.00 | 8 | 0.33 | 4 | 0.32 | 2 | 1.00 | 4 | 0.32 | 3 | 0.56 | 1 | - | 3 | 0.32 |
|  | tumor | 12 | 1.00 | 8 | 0.32 | 14 | **0.024** | 14 | 1.00 | 8 | 0.083 | 12 | 0.084 | 14 | 1.00 | 9 | 0.18 |
| 96h delay | normal | 1 | - | 2 | 1.00 | 1 | - | 0 | - | 1 | - | 0 | - | 0 | - | 0 | - |
|  | tumor | 10 | 1.00 | 6 | 0.11 | 11 | 0.12 | 7 | 0.65 | 6 | 0.26 | 8 | 0.32 | 10 | 1.00 | 4 | 0.65 |
| 2 days fixation | normal | 5 | 1.00 | 4 | 1.00 | 5 | 1.00 | 3 | 1.00 | 6 | 1.00 | 6 | 0.71 | 3 | 1.00 | 1 | - |
|  | tumor | 13 | 1.00 | 11 | 0.16 | 13 | 0.10 | 8 | 0.18 | 11 | 0.56 | 12 | 1.00 | 13 | 1.00 | 13 | 0.32 |
| 4 days fixation | normal | 4 | 1.00 | 6 | 0.16 | 7 | 1.00 | 4 | 1.00 | 7 | 0.083 | 7 | 0.71 | 3 | 1.00 | 2 | 1.00 |
|  | tumor | 14 | 1.00 | 10 | 1.00 | 11 | 0.32 | 8 | 0.18 | 11 | 0.89 | 13 | 1.00 | 10 | 1.00 | 12 | 0.32 |
| 7 days fixation | normal | 4 | 1.00 | 4 | 0.32 | 6 | 1.00 | 4 | 1.00 | 7 | 0.083 | 6 | 1.00 | 3 | 1.00 | 4 | 1.00 |
|  | tumor | 14 | 1.00 | 11 | 0.32 | 12 | 0.32 | 10 | 0.18 | 11 | 0.16 | 14 | 0.16 | 12 | 1.00 | 12 | 1.00 |

n=number of available cores with IHC scores 1 to 4 in both fixations.

| **Sample** | **Tissue** | **PDL-1** | | **PL-1 (%tumor cells)** | | **ROS** | | **CMET** | | **P63** | | **CK 5/6** | | **Napsin A** | | **D2-40** | | **TTF1 (Ventana)** | |
| --- | --- | --- | --- | --- | --- | --- | --- | --- | --- | --- | --- | --- | --- | --- | --- | --- | --- | --- | --- |
|  |  | **n** | **p-value** | **n** | **p-value** | **n** | **p-value** | **n** | **p-value** | **n** | **p-value** | **n** | **p-value** | **n** | **p-value** | **n** | **p-value** | **n** | **p-value** |
| 1h delay | normal | 16 | **0.008** |  |  | 3 | 1.00 | 5 | 0.16 | 0 | - | 20 | 0.18 | 20 | 0.89 | 20 | 0.56 | 6 | 1.00 |
|  | tumor | 12 | 0.40 | 19 | 0.091 | 11 | 1.00 | 13 | 0.71 | 12 | 0.32 | 20 | 0.48 | 18 | 0.17 | 18 | 0.32 | 13 | 1.00 |
| 6h delay | normal | 17 | **0.021** |  |  | 3 | 1.00 | 6 | **0.046** | 0 | - | 19 | 0.059 | 19 | 0.68 | 19 | 0.65 | 6 | 0.32 |
|  | tumor | 15 | 0.47 | 19 | 0.17 | 16 | 1.00 | 16 | 0.10 | 15 | 0,41 | 19 | 0.19 | 17 | 0.26 | 18 | 1.00 | 16 | 0.16 |
| 24h delay | normal | 14 | 0.070 |  |  | 2 | 1.00 | 3 | 0.16 | 0 | - | 19 | 1.00 | 20 | **0.045** | 20 | **0.041** | 4 | 0.32 |
|  | tumor | 10 | 0.11 | 19 | 0.062 | 15 | 1.00 | 12 | **0.020** | 13 | 0.46 | 17 | 0.26 | 18 | **0.023** | 18 | 0.32 | 12 | 0.18 |
| 48h delay | normal | 13 | 0.086 |  |  | 3 | 1.00 | 3 | 0.10 | 0 | - | 18 | 0.18 | 20 | 0.47 | 19 | **0.044** | 4 | 0.32 |
|  | tumor | 11 | 0.063 | 19 | 0.074 | 13 | 1.00 | 10 | **0.034** | 11 | 0.16 | 18 | 0.12 | 19 | **0.023** | 17 | 0.32 | 13 | 0.059 |
| 96h delay | normal | 11 | **0.004** |  |  | 2 | 1.00 | 1 | - | 0 | - | 16 | 1.00 | 20 | **0.023** | 15 | **0.026** | 4 | 0.16 |
|  | tumor | 9 | 0.066 | 19 | **0.017** | 10 | 1.00 | 11 | **0.038** | 6 | 0.59 | 16 | 0.14 | 18 | **0.030** | 16 | 0.32 | 15 | **0.031** |
| 2 days fixation | normal | 14 | 0.23 |  |  | 2 | 1.00 | 4 | 0.56 | 1 | - | 20 | 0.32 | 19 | 0.12 | 20 | 0.87 | 5 | 0.32 |
|  | tumor | 11 | 0.32 | 12 | 0.28 | 10 | 1.00 | 12 | 0.26 | 11 | 0.32 | 19 | 0.56 | 17 | 1.00 | 19 | 0.32 | 14 | 1.00 |
| 4 days fixation | normal | 11 | 0.65 |  |  | 5 | 1.00 | 5 | 0.32 | 1 | - | 20 | 0.16 | 18 | 0.29 | 20 | 0.48 | 7 | 1.00 |
|  | tumor | 9 | 0.16 | 12 | 0.59 | 14 | 1.00 | 11 | 0.32 | 11 | 0.18 | 19 | 0.81 | 17 | 0.083 | 18 | 0.32 | 15 | 0.56 |
| 7 days fixation | normal | 14 | 0.10 |  |  | 5 | 0.32 | 2 | 0.32 | 0 | - | 19 | 1.00 | 19 | 0.20 | 19 | 0.26 | 9 | 0.32 |
|  | tumor | 12 | 1.00 | 10 | 1.00 | 14 | 1.00 | 12 | 0.71 | 12 | 1.00 | 18 | 1.00 | 14 | 0.32 | 18 | 0.32 | 14 | 0.65 |

| **Sample** | **Tissue** | **ALK D5F3** | | **CK7** | | **EGFR (Dako)** | | **PD-L1 (22c3)** | | **PL-1 (%tumor cells)** | | **TTF1 (Dako)** | | **SINAPTO-PHISIN** | | **CHROMOGRANIN** | | **CD 56** | |
| --- | --- | --- | --- | --- | --- | --- | --- | --- | --- | --- | --- | --- | --- | --- | --- | --- | --- | --- | --- |
|  |  | **n** | **p-value** | **n** | **p-value** | **n** | **p-value** | **n** | **p-value** | **n** | **p-value** | **n** | **p-value** | **n** | **p-value** | **n** | **p-value** | **n** | **p-value** |
| 1hr delay | normal | 20 | 1.00 | 4 | 0.32 | 4 | 1.00 |  |  |  |  | 1 | - | 7 | 1.00 | 8 | 1.00 | 9 | 1,00 |
|  | tumor | 20 | 1.00 | 12 | 0.19 | 12 | 0.32 | 14 | 0.56 | 1 | - | 2 | 0.32 | 7 | 1.00 | 8 | 1.00 | 9 | 1,00 |
| 6hrs delay | normal | 20 | 1.00 | 5 | 1.00 | 2 | 1.00 |  |  |  |  | 2 | 0.32 | 13 | 1.00 | 12 | 1.00 | 10 | 1,00 |
|  | tumor | 20 | 1.00 | 13 | 0.066 | 14 | 1.00 | 14 | 0.48 | 2 | 1.00 | 3 | 0.65 | 13 | 1.00 | 12 | 0.32 | 10 | 1,00 |
| 24hrs delay | normal | 20 | 1.00 | 3 | 1.00 | 0 | - |  |  |  |  | 1 | - | 9 | 1.00 | 12 | 1.00 | 6 | 1,00 |
|  | tumor | 20 | 1.00 | 11 | 0.28 | 11 | 0.67 | 10 | 1.00 | 2 | 0.65 | 2 | 0.32 | 9 | 0.18 | 11 | 0.32 | 6 | 1,00 |
| 48hrs delay | normal | 20 | 1.00 | 5 | 1.00 | 3 | 1.00 |  |  |  |  | 2 | 0.32 | 10 | 0.32 | 15 | 1.00 | 11 | 1,00 |
|  | tumor | 20 | 1.00 | 13 | 0.071 | 12 | 0.27 | 14 | 0.56 | 3 | 0.65 | 3 | 0.32 | 10 | 0.18 | 15 | 0.18 | 11 | 0,10 |
| 96 hrs delay | normal | 20 | 1.00 | 4 | 0.18 | 0 | - |  |  |  |  | 0 | - | 7 | 1.00 | 7 | 1.00 | 4 | 1,00 |
|  | tumor | 20 | 1.00 | 8 | 0.18 | 11 | 0.25 | 12 | 1.00 | 2 | 0.32 | 2 | 1.00 | 7 | 1.00 | 6 | 1.00 | 4 | 1,00 |
| 2 days fixation | normal | 20 | 1.00 | 6 | 1.00 | 6 | 1.00 |  |  |  |  | 4 | 1.00 | 9 | 0.32 | 10 | 1.00 | 10 | 1,00 |
|  | tumor | 20 | 1.00 | 14 | 0.32 | 12 | 1.00 | 11 | 0.32 | 4 | 1.00 | 6 | 1.00 | 9 | 0.32 | 10 | 1.00 | 10 | 1,00 |
| 4 days fixation | normal | 20 | 1.00 | 8 | 1.00 | 5 | 0.32 |  |  |  |  | 4 | 1.00 | 11 | 1.00 | 11 | 1.00 | 10 | 1,00 |
|  | tumor | 20 | 1.00 | 14 | 0.066 | 13 | 0.21 | 13 | 0.56 | 4 | 0.41 | 7 | 1.00 | 11 | 1.00 | 11 | 1.00 | 10 | 1,00 |
| 7 days fixation | normal | 20 | 1.00 | 8 | 1.00 | 7 | 0.65 |  |  |  |  | 5 | 1.00 | 9 | 0.32 | 11 | 1.00 | 11 | 1,00 |
|  | tumor | 20 | 1.00 | 14 | 0.10 | 12 | 0.79 | 12 | 0.32 | 2 | 0.65 | 6 | 1.00 | 9 | 0.32 | 11 | 1.00 | 11 | 1,00 |
