# Supplementary material for: Impact of delayed and prolonged fixation on the evaluation of immunohistochemical staining on lung carcinoma resection specimen
Source: Virchows Arch. 2019 Jul 1;475(2):191–9. doi: 10.1007/s00428-019-02595-9 (PMC6647403; doi:10.1007/s00428-019-02595-9)
Supplement: Supplementary file 5 — (DOCX 19 kb) [file 428_2019_2595_MOESM5_ESM.docx]

Supplementary table 5 *Distribution of tumor cell PD-L1 percentages of the 20 cores/sample. Each separate TMA for delayed and prolonged fixation contained a different standard fixated sample.*

| **% tumorcel** | **Standard fixation** | | **1h delay** | | **6h delay** | | **24h delay** | | **48h delay** | | **96h delay** | | **Standard fixation** | | **2 days fixation** | | **4 days fixation** | | **7 days fixation** | |
| --- | --- | --- | --- | --- | --- | --- | --- | --- | --- | --- | --- | --- | --- | --- | --- | --- | --- | --- | --- | --- |
|  | **n** | **%** | **n** | **%** | **n** | **%** | **n** | **%** | **n** | **%** | **n** | **%** | **n** | **%** | **n** | **%** | **n** | **%** | **n** | **%** |
| 0 | 11 | 55% | 17 | 85% | 15 | 75% | 17 | 85% | 17 | 85% | 18 | 90% | 15 | 75% | 9 | 45% | 9 | 45% | 10 | 50% |
| 1-5% | 1 | 5% | 0 | 0% | 2 | 10% | 0 | 0% | 0 | 0% | 0 | 0% | 0 | 0% | 0 | 0% | 0 | 0% | 0 | 0% |
| 5-10% | 0 | 0% | 0 | 0% | 1 | 5% | 0 | 0% | 0 | 0% | 0 | 0% | 0 | 0% | 0 | 0% | 2 | 10% | 1 | 5% |
| 10-25% | 3 | 15% | 1 | 5% | 0 | 0% | 0 | 0% | 0 | 0% | 0 | 0% | 0 | 0% | 2 | 10% | 1 | 5% | 1 | 5% |
| 25-50% | 1 | 5% | 0 | 0% | 1 | 5% | 1 | 5% | 0 | 0% | 1 | 5% | 0 | 0% | 1 | 5% | 2 | 10% | 1 | 5% |
| >50% | 3 | 15% | 2 | 10% | 1 | 5% | 2 | 10% | 3 | 15% | 1 | 5% | 1 | 5% | 2 | 10% | 1 | 5% | 1 | 5% |
| missing | 1 | 5% | 0 | 0% | 0 | 0% | 0 | 0% | 0 | 0% | 0 | 0% | 4 | 20% | 6 | 30% | 5 | 25% | 6 | 30% |
