# Supplementary material for: Impact of delayed and prolonged fixation on the evaluation of immunohistochemical staining on lung carcinoma resection specimen
Source: Virchows Arch. 2019 Jul 1;475(2):191–9. doi: 10.1007/s00428-019-02595-9 (PMC6647403; doi:10.1007/s00428-019-02595-9)

**Supplementary fig. 1** Cross section of lobectomy cut for first time after 40 hours fixation showing peripherally fixed (grey) zone and a centrally unfixed area (red).


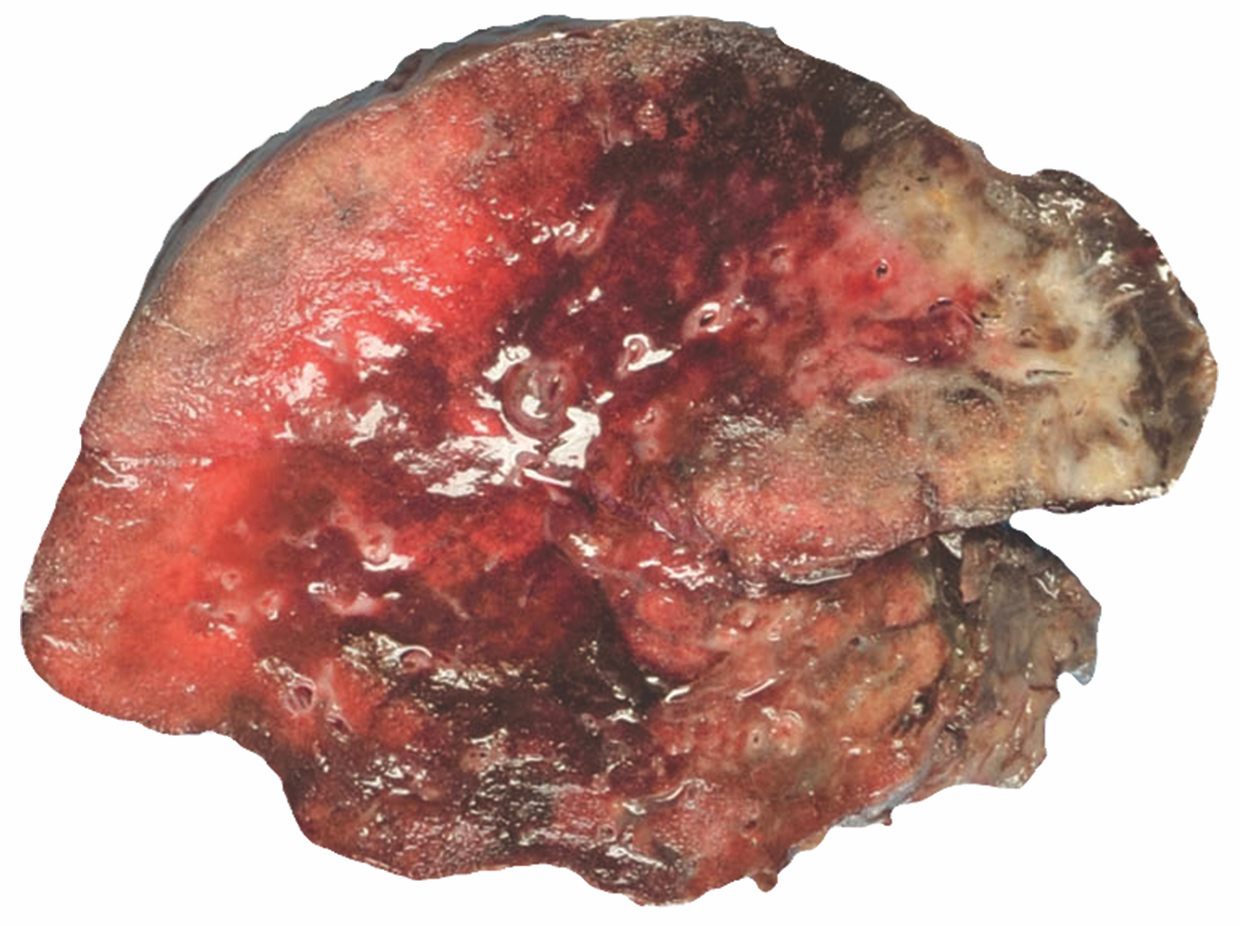

Supplement: Supplementary file 6 — (DOCX 1466 kb) [file 428_2019_2595_MOESM6_ESM.docx]
